# Organelle Genome Variation in the Red Algal Genus *Ahnfeltia* (Florideophyceae)

**DOI:** 10.3389/fgene.2021.724734

**Published:** 2021-09-27

**Authors:** Hocheol Kim, Ji Hyun Yang, Danilo E. Bustamante, Martha S. Calderon, Andres Mansilla, Christine A. Maggs, Gayle I. Hansen, Hwan Su Yoon

**Affiliations:** ^1^ Department of Biological Sciences, Sungkyunkwan University, Suwon, South Korea; ^2^ Instituto de Investigación para el Desarrollo Sustentable de Ceja de Selva (INDES-CES), Universidad Nacional Toribio Rodríguez de Mendoza, Chachapoyas, Peru; ^3^ Laboratorio de Macroalgas Antárticas y Subantárticas, Universidad de Magallanes, Punta Arenas, Chile; ^4^ School of Biological Sciences, Queen’s University Belfast, Belfast, United Kingdom; ^5^ Marine Algal Biodiversity Research, Newport, OR, United States

**Keywords:** plastid genome, mitochondrial genome, genome architecture variation, *Ahnfeltia*, genetic diversity, phylogeograhy

## Abstract

The agarophyte *Ahnfeltia* (Ahnfeltiales, Rhodophyta) is a globally widespread genus with 11 accepted species names. Two of the most widespread species in this genus, *A. plicata* and *A. fastigiata*, may have diverged genetically due to past geographic changes and subsequent geographic isolation. To investigate this genomic and genetic diversity, we generated new plastid (ptDNAs) and mitochondrial genomes (mtDNAs) of these *Ahnfeltia* species from four different regions (*A. plicata* - Chile and UK and *A. fastigiata* - Korea and Oregon). Two architecture variations were found in the *Ahnfeltia* genomes: in ptDNA of *A. fastigiata* Oregon, the hypothetical pseudogene region was translocated, likely due to recombination with palindromic repeats or a gene transfer from a red algal plasmid. In mtDNA of *A. fastigiata* Korea, the composition of the group II intronic ORFs was distinct from others suggesting different scenarios of gain and loss of group II intronic ORFs. These features resulted in genome size differences between the two species*.* Overall gene contents of organelle genomes of *Ahnfeltia* were conserved. Phylogenetic analysis using concatenated genes from ptDNAs and mtDNAs supported the monophyly of the Ahnfeltiophycidae. The most probable individual gene trees showed that the *Ahnfeltia* populations were genetically diversified. These trees, the *cox*1 haplotype network, and a dN/dS analysis all supported the theory that these *Ahnfeltia* populations have diversified genetically in accordance with geographic distribution.

## Introduction

The Florideophyceae is a species-rich (ca. 6,700 spp.) red algal class that has been divided into five subclasses: the Hidenbrandiophycidae, Nemaliophycidae, Corallinophycidae, Rhodymeniophycidae, and Ahnfeltiophycidae (Yoon et al., 2016). Among the five subclasses, the Ahnfeltiophycidae are unique in having a heteromorphic life cycle and cylindrical pit plugs without cap layers (Maggs and Pueschel, 1989). In molecular phylogenies, this subclass is the closest sister group to the taxon-rich Rhodymeniophycidae (Yang et al., 2016; Yoon et al., 2016), but the Ahnfeltiophycidae consists of only two genera: *Ahnfeltia* and the recently added microscopic *Pihiella* (Huisman et al., 2003; Kamiya et al., 2017; Guiry and Guiry, 2021). Species of *Ahnfeltia* live on intertidal and shallow sublittoral bedrock and form tangled branching tufts. Their thin and wiry branches are dark red or dark brown in color and divide in an irregularly dichotomous pattern (Rayment, 2004).

*Ahnfeltia*, the subject of this study, is commercially important as it is a valuable agarophyte, known for high-quality agar production with a low sulfate ratio (Chapman and Chapman, 1980; Truus et al., 2006; Zhang et al., 2019). The sulfate ratio of agar from *Ahnfeltia plicata* is less than half of that of the well-known agarophytes, *Gelidium amansii* and *Gracilariopsis lemaneiformis* (Zhang et al., 2019). Commercial production of agar from *Ahnfeltia* occurs in both Russia and Japan, and the agar is known as Itani agar in Japan and Sakhalin agar in Russia. In particular, 170,000–230,000 wet tons of *Ahnfeltia* are reported to be harvested annually in Russia (Titlyanov et al., 1999; Truus et al., 2006).

Allopatric divergence and speciation in populations is known to occur when geographic barriers such as mountains, rivers, and oceans block gene flow causing reproductive isolation. Numerous phylogeographic studies on macroalgal species have shown that divergence of these marine organisms is related to past climate and tectonic changes (Maggs et al., 2008; Yang et al., 2009; Boo et al., 2019; Bringloe et al., 2019; Yang et al., 2020). Repeated glaciation and deglaciation during the glacial periods are considered to be one of the main factors for macroalgal divergence (Maggs et al., 2008; Bringloe et al., 2019). For example, Bringloe and Saunders (2019) found that the trans-Arctic speciation of several red algae, including *Ahnfeltia,* occurred following range extensions from the North Pacific to the North Atlantic during the Pleistocene after the opening of the Bering Strait.

Past geographic events and subsequent geographic isolation during the glacial period may also have accelerated the genomic and genetic divergence of *Ahnfeltia*. The Ahnfeltiophycidae is estimated to have diverged around 500 million years ago (Mya) and speciation events took place 2-5 Mya (Yang et al., 2016; Bringloe and Saunders, 2019). *Ahnfeltia plicata* is a cold temperate species distributed in both the North Atlantic and the South Pacific including Australia and Chile (Maggs and Pueschel, 1989; Milstein and Saunders, 2012). After deglaciation, the equator appears to have acted as a geographic barrier since high temperatures are not favorable for the growth of *Ahnfeltia* (Lüning and Freshwater, 1988; Mansilla et al., 2014). *Ahnfeltia fastigiata* is distributed along the coasts of the Northeast (NE) and Northwest (NW) Pacific (Lee and Kang, 2001; Milstein and Saunders, 2012). In East Asia, several places appear to be macroalgal refugia, isolated due to tectonic events and changes in current circulation patterns after glacial periods (Yang et al., 2009; Hu et al., 2011; Boo et al., 2019; Yang et al., 2020). This may have affected the genomic diversity of *A. fastigiata* in these areas.

Studies that cover population genomics, taxonomy, phylogenomics, and evolution often require large datasets. Recently, these have been greatly facilitated by the methods used for next generation sequencing (NGS) since these methods can examine and analyze extensive arrays of data. This is particularly useful for data obtained from organelle genomes (Smith and Keeling, 2015). In previous studies, mitochondrial (Yang et al., 2015) and plastid (Lee et al., 2016a) genomes from a number of diverse red algal orders were assembled and used to elucidate the complete evolutionary history of these lineages. However, only one plastid genome (ptDNA—NC_031145) and one mitochondrial genome (mtDNA—NC_026054) of *A. plicata* from Chile have been available in the NCBI database (Yang et al., 2015; Lee et al., 2016a). In order to add to this dataset and begin to test our geographic theories, we generated new ptDNAs and mtDNAs for both *A. plicata* and *A. fastigiata*, each from two different remote geographic regions. This gave us the opportunity to analyze and evaluate the influence of geographic isolation on the genomic and genetic divergence of *Ahnfeltia* organelle genomes.

## Materials and Methods

### Sample Collection and Preparation

**FIGURE 1:**
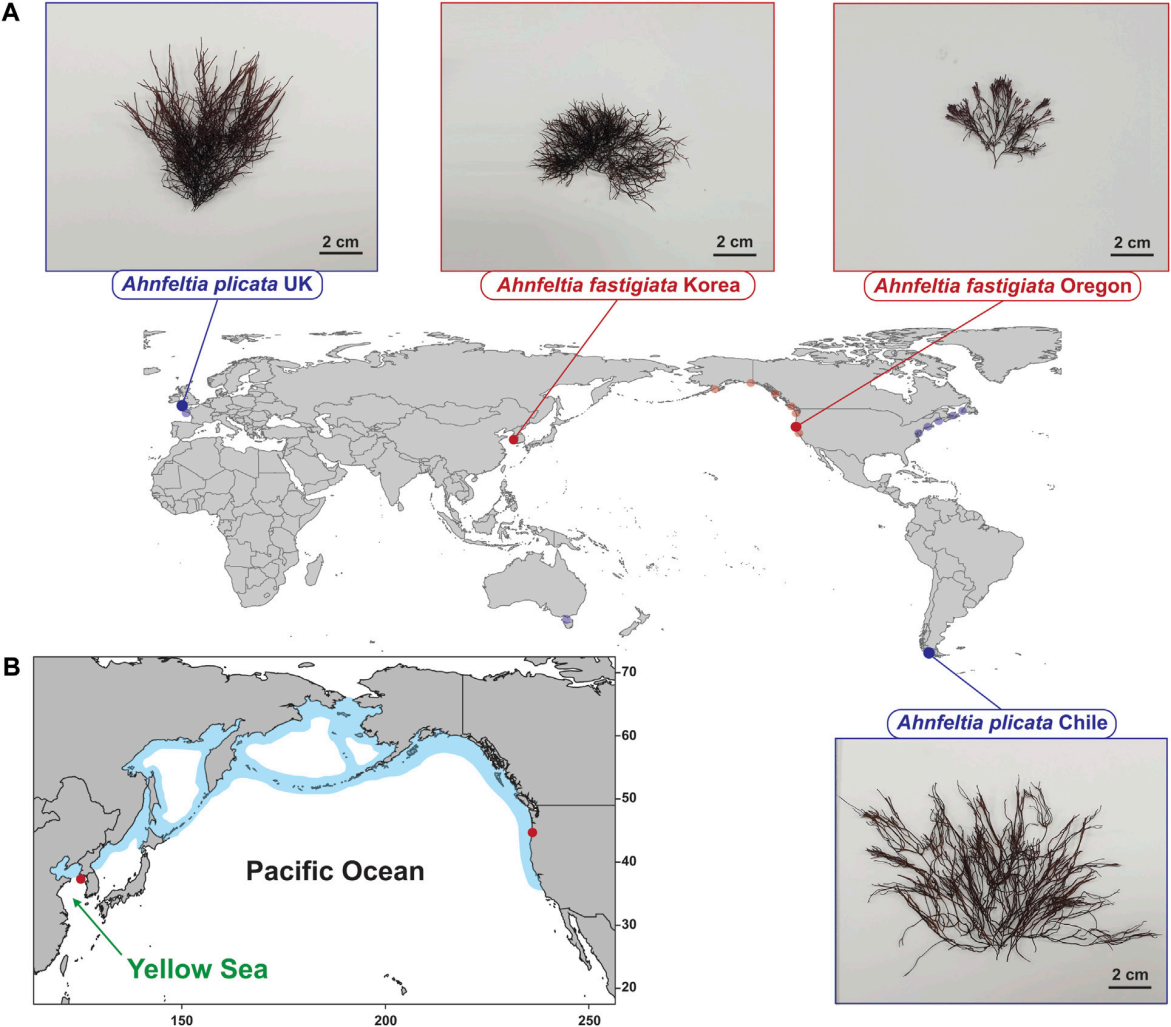
**(A)** Collection sites of *Ahnfeltia*. *A. plicata* were collected in Seno Skyring, Magallanes, Chile and Freshwater West, Pembrokeshire, Wales, UK to compare the genetic and genomic diversity between the Northern hemisphere and Southern hemisphere strains. *A. fastigiata* were collected in Baeknyeong island, Korea and Seal Rock, Oregon, USA to compare the NE Pacific and the NW Pacific strains. Translucent dots represent sampling sites of *rbc*L and *cox*1 data from NCBI database and Milstein and Saunders’ study (2012) that were used for *rbc*L phylogeny and *cox*1 haplotype network in this study. **(B)** Distribution of *A. fastigaiata* based on Algaebase information (Guiry and Guiry, 2021).

Four populations of fresh *Ahnfeltia* thalli were sampled by snorkeling or collecting from tidepools in intertidal zones in the following regions. *A. plicata* populations were sampled from Seno Skyring, Magallanes, Chile (52°32′45.1″S 71°56′46.3″W), and Freshwater West, Pembrokeshire, Wales, UK (51°39′35.9″N 5°03′52.2″W). *A. fastigiata* samples were collected from Baeknyeong island in Yellow Sea, Republic of Korea (37°59′01.3″N 124°41′54.0″E), and Seal Rock, Oregon, USA (44°29′48.5″N 124°05′07.5″W) (Figure 1A). The collected samples were placed in seawater in plastic bags or 50 ml conical tubes (SPL Life Sciences, Gyeonggi-do, South Korea) and stored in an icebox for transfer to the laboratory. Attached eukaryote contaminants such as small barnacles and other epiphytes were manually removed with forceps. Samples were then wiped using a brush and washed with sterilized seawater to prevent further contamination.

### DNA Extraction and Species Identification Using *rbc*L Phylogeny

Total genomic DNAs of each population were extracted with a modified cetyltrimethylammonium bromide (CTAB) protocol (Doyle and Doyle., 1987). To confirm the species, *rbc*L genes were determined before genome sequencing. The *rbc*L marker, which encodes the ribulose-1,5-bisphosphate carboxylase large subunit, is the most widely used in species identification in the red algae (Saunders and Moore, 2013). The *rbc*L primers were F57 and *rbc*LrevNew described in Freshwater and Rueness (1994) and Saunders and Moore (2013). Amplified DNAs were purified using the LaboPass™ PCR Purification Kit (COSMO Genetech, Seoul, Republic of Korea). PCR products were sent for sequencing to Macrogen (Seoul, Republic of Korea). After a quality check, the forward and reverse sequences were merged using Geneious prime 2020.0.4 (https://www.geneious.com). For phylogenetic analysis, published *rbc*L sequences of Rhodymeniophycidae and *Ahnfeltia* were obtained from NCBI (see accession number of each taxon in Supplementary Table S1), and alignments for all sequences were conducted with MAFFT v7.310 (Katoh and Standley, 2013). A maximum likelihood (ML) phylogenetic tree was reconstructed by IQ-TREE v.1.6.8 with 1,000 ultrafast bootstrap replications (-bb 1000) and model test option (-m TEST) (Nguyen et al., 2015).

### Whole Genome Sequencing, Genome Assembly, and Gene Annotation

Approximately 30 μg of genomic DNA from each population were sent to DNA-link Inc. (Seoul, Republic of Korea) for whole genome sequencing. PacBio Sequel I (Pacific Biosciences, Inc., Menlo Park, CA, USA) and Illumina Nova Seq 6000 (Illumina, San Diego, USA) platforms were used to generate long- and short-read raw data, respectively. Organelle genome assembly for each population was conducted with two methods using short and long reads data. Before using Illumina data, low-quality reads were eliminated using Trimmomatic v0.39 (Bolger et al., 2014). NOVOPlasty assembler was used for organelle assembly using short reads (Dierckxsens et al., 2017). Flye assembler was used for long reads (Kolmogorov et al., 2019). To complete partially assembled organelle genomes and check the accuracy of assembled organelle genomes, initial assembled genomes and contigs from NOVOPlasty were compared with results from Flye assembler using Geneious prime 2020.0.4 (https://www.geneious.com). Assembled genomes were corrected by Pilon (Walker et al., 2014). Primary gene annotation was conducted on GeSeq server, which provides the annotation for organelle genomes based on published genomes (Tillich et al., 2017). Protein coding genes (CDS) were manually double-checked by blasting primary annotated CDSs on NCBI and comparing them to CDSs of published ptDNA (NC_031145) and mtDNA (NC_026054) of *A. plicata* (Yang et al., 2015; Lee et al., 2016a). Annotated tRNAs were double-checked on the ARAGORN website (Laslett and Canback, 2004). rRNAs were searched on the RNAmmer 1.2 website and compared with published genomes (Lagesen et al., 2007).

### Genome Correction for ptDNA of *A. plicata* NC_031145

Both short and long read assembly results showed that the direction of the small single copy (SSC) region of ptDNAs was opposite to the published ptDNA of *A. plicata* (NC_031145). Because the published *A. plicata* ptDNA was based on a collection from Magallanes, Chile, and generated based on the short-read Ion Torrent PGM platform (Lee et al., 2016a) by this research group, we investigated an assembly error in the published *A. plicata* ptDNA (NC_031145). The direction of the SSC region was confirmed by PCR and Sanger sequencing. Specific primers were manually produced using Geneious prime 2020.0.4 (https://www.geneious.com). Detailed information on PCR locations and primers for IR regions is provided in the supplementary information (Supplementary Table S2). PCRs were carried out using the KOD DNA polymerase (TOYOBO, Osaka, Japan). Isoforms that are different in the direction of the SSC region between inverted regions (IRs) caused by flip-flop recombination have been reported in the ptDNA of green algae and land plants (Stein et al., 1986; Day and Madesis, 2007; Turmel et al., 2017; Kim et al., 2019). However, we were not able to find “flip-flop” forms in *Ahnfeltia* (Supplementary Figure S1).

### Phylogenetic Analyses

To construct phylogenetic trees with concatenated genes from organelle genomes, ptDNAs and mtDNAs genes were collected from *Ahnfeltia* genomes as well as all available organelle genomes of Florideophyceae and Bangiophyceae as an outgroup (see accession number for each genome in Supplementary Tables S6, S7). 130 plastid protein coding genes from 149 ptDNAs and 11 mitochondrial protein coding genes from 103 mtDNAs were used for the phylogenomic analysis following a method described in Lee et al. (2016a). Translated protein sequences of genes were aligned with MAFFT v7.310 (Katoh and Standley, 2013). ML phylogenetic trees were constructed with IQ-TREE v1.6.8 with 1,000 ultrafast bootstrap replications (-bb 1000) and the test model (-m TEST) (Nguyen et al., 2015).

Since different compositions of group II intronic ORFs in mtDNAs were found in the *Ahnfeltia* populations, we conducted a phylogenetic analysis with ML phylogenetic trees to find the origin. Homologous group II intronic ORFs were obtained from the NCBI protein database (nrDB) using MMseqs2 (Steinegger and Söding, 2017). Mitochondrial group II intronic ORFs were aligned using MAFFT v7.310 (Katoh and Standley, 2013). ML trees were constructed with IQ-TREE v1.6.8 with 1,000 ultrafast bootstrap replications (-bb 1000) and the test model option (-m TEST) (Nguyen et al., 2015).

### Plastid and Mitochondrial Genome Analyses

Assembled organelle genome maps were drawn using the OGDRAW v1.3.1 server (Greiner et al., 2019). Results of OGDRAW were manually modified to clearly compare locations of group II intronic ORFs in mtDNAs. The comparison of ptDNAs was conducted with the progressiveMauve algorithm of Mauve v2.3.1 (Darling et al., 2010). The visualization of backbone files and tree files was performed using genoplotR (Guy et al., 2010). Tandem repeat analysis was conducted on the REPuter server (Kurtz et al., 2001). Tandem repeats were collected over 20 base pairs. The visualization of repeats with assembled ptDNAs was conducted with CIRCOS (Krzywinski et al., 2009) and DNAplotter (Carver et al., 2009). The red algal plasmid network was constructed using EGN (Halary et al., 2013) and Cytoscape (Shannon et al., 2003) with results of BLASTx searches for hypothetical genes in *A. plicata* and hypothetical pseudogenes in *A. fastigiata*.

### dN/dS Analysis

Non-synonymous substitutions (dN), synonymous substitutions (dS), and dN/dS ratio were calculated to estimate the evolutionary selection pressure on CDSs in organelle genomes of *Ahnfeltia*. CDSs from each genome were extracted using a python script. These were then aligned using MAFFT v7.310 (Katoh and Standley, 2013). Aligned fasta files were converted to AXT files using a perl script (parseFastaIntoAXT.pl), and KaKs calculator 2.0 was used for dN, dS, and dN/dS calculation (Wang et al., 2010). Calculated values were plotted using ggplot2 in R (Wickham, 2016). Outliers in boxplots were eliminated following the IQR rule.

### Evaluation of Gene Tree Conflicts and *cox*1 Haplotype Network

To check for incomplete lineage sorting and gene tree conflict, we compared all possible individual gene trees from ptDNAs and mtDNAs with the species tree from the concatenated gene dataset. Based on Smith et al. (2015), basic steps were followed as Matt Johnson described at a Kew Royal Botanic Gardens workshop (https://github.com/mossmatters/KewHybSeqWorkshop). All individual gene trees were first constructed with the outgroup Rhodymeniophycidae using IQ-TREE v1.6.8 with 1,000 fast bootstrap replications (-b 1000) and model test option (-m TEST) (Nguyen et al., 2015). A coalescent tree was generated using ASTRAL-II (Mirarab and Warnow, 2015) and pie charts were constructed using phyparts (Smith et al., 2015). Lastly, gene trees were cut off if they had under 80% bootstrap support.

We also constructed a *cox*1 haplotype network to evaluate genetic variation based on geographic distribution. We compared *cox*1 sequences of the four new *Ahnfeltia* populations with the COI-5P results of Milstein and Saunders (2012), which used 99 sequences of *A. plicata* and 30 sequences of *A. fastigiata*. Alignment was conducted with MAFFT v7.310 (Katoh and Standley, 2013), and the haplotype network was constructed using PopART with integer neighbor-joining networks (Leigh and Bryant, 2015).

## Results

Our studies revealed a wide array of similarities and differences between the species and the geographic populations.

### General Feature of ptDNAs

The genome sizes of the four assembled ptDNAs of *Ahnfeltia* were 191–194 kb. Specifically, the ptDNA of *A. plicata* Chile was 191,622 bp, *A. plicata* UK was 191,647 bp, *A. fastigiata* Korea was 194,202 bp, and *A. fastigiata* Oregon was 193,253 bp. The ptDNAs of *A. fastigiata* were larger than those of *A. plicata* (Table 1). Overall GC contents of each genome were almost the same: 32.2–32.7%. The number of rRNA, tmRNA, and introns were also the same: 6, 1, and 1, respectively. There were 205 and 204 CDSs in *A. plicata* and *A. fastigiata,* respectively, the difference being the result of having one additional hypothetical protein in *A. plicata,* which was pseudogenized with a nonsense mutation in *A. fastigiata*. There were 33 tRNAs in *A. plicata* Chile, *A. plicata* UK, and *A. fastigiata* Korea but 32 in *A. fastigiata* Oregon due to one missing tRNA (trnI-GAU) between 16S rRNA (rrn16) and 23S rRNA (rrn23) in an inverted repeat region.

**TABLE 1:** General genomic feature of ptDNAs and mtDNAs of *Ahnfeltia.*

	ptDNA					mtDNA				
Species	*Ahnfeltia fastigiata*	*Ahnfeltia fastigiata*	*Ahnfeltia plicata*	*Ahnfeltia plicata*	*Ahnfeltia plicata*	*Ahnfeltia fastigiata*	*Ahnfeltia fastigiata*	*Ahnfeltia plicata*	*Ahnfeltia plicata*	*Ahnfeltia plicata*
Collection Site	Korea	Oregon	UK	Chile	Chile	Korea	Oregon	UK	Chile	Chile
Genome Size (bp)	194,202	193,253	191,647	191,622	190,451	33,492	33,309	32,819	32,868	32,878
GC %	32.4	32.2	32.7	32.7	32.5	32.9	33.0	33.4	33.4	33.4
CDSs	204	204	205	205	205	25	26	26	26	26
rRNA	6	6	6	6	6	2	2	2	2	2
tRNA	33	32	33	33	31	24	24	24	24	24
tmRNA	1	1	1	1	1	-	-	-	-	-
Intron	1	1	1	1	1	3	3	3	3	3
NCBI Accession	MZ393455 (This study)	MZ393454 (This study)	MZ393452 (This study)	MZ393453 (This study)	NC_031145	MZ393456 (This study)	MZ393457 (This study)	MZ393459 (This study)	MZ393458 (This study)	NC_026054

### Translocation and Genome Expansion in ptDNAs of *A. fastigiata*

**FIGURE 2:**
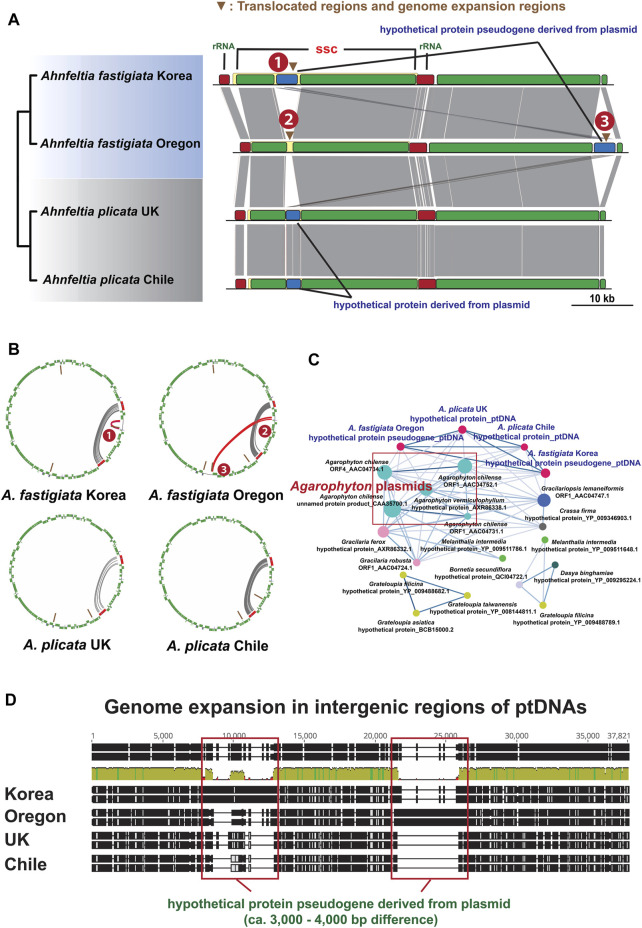
**(A)** Mauve alignment of ptDNAs of *Ahnfeltia*. Hypothetical protein pseudogene region in SSC region was translocated in *A. fastigiata* Oregon (From 1 to 3) and genome expansion also occurred in this region. **(B)** Tandem repeat analysis results and circular plot showing that tandem repeats were mainly accumulated in inverted ribosomal operon regions (IRs; Grey lines) as well as in the translocated regions of *A. fastigiata* (Red lines). **(C)** Cytoscape network of hypothetical protein genes in *A. plicata* and hypothetical protein pseudogene in *A. fastigiata*. All genes and pseudogene had a homology with red algal plasmids such as *Agarophyton* plasmids. **(D)** Alignments of intergenic regions of ptDNAs showing genome extended regions. About 3,000–4,000 base pairs were different around hypothetical protein pseudogene regions in ptDNAs, which made the ptDNA sizes of *A. fastigiata* larger than those of *A. plicata*.

Plastid genome architectures of the *Ahnfeltia* samples were almost identical, but one regional difference was found in *A. fastigiata* Oregon. In the Mauve whole genome alignment, the hypothetical protein pseudogene was translocated to the LSC region in *A. fastigiata* Oregon but was located in the SSC region in other strains. Figure 2A shows the comparative ptDNA structure with the translocations from the original position 1 to the new position 3 in *A. fastigiata* Oregon. In addition, genome expansion mainly occurred around the hypothetical protein pseudogene regions in *A. fastigiata* (Figures 2A, D). Palindromic tandem repeats were accumulated around hypothetical protein pseudogene regions in *A. fastigiata* (Figure 2B). Sequences from this region were homologous with red algal plasmids and they were particularly close to *Agarophyton* plasmids (Figure 2C).

### General Feature of mtDNAs

The mtDNAs size of *Ahnfeltia* were 32–33 kb: 32,868 bp (*A. plicata* Chile), 32,819 bp (*A. plicata* UK), 33,492 bp (*A. fastigiata* Korea), and 33,309 bp (*A. fastigiata* Oregon). The number of CDSs was 26 in *A. plicata* Chile, *A. plicata* UK, and *A. fastigiata* Oregon, but there were only 25 CDSs in *A. fastigiata* Korea due to a pseudogenized intronic ORF in *cox*1 (*orf780* in this study). GC contents were very similar, 32.9–33.4%. The numbers of rRNA, tRNA, and introns were the same in all taxa, 2, 24, and 3, respectively (Table 1).

### Different Group II Intron Composition in mtDNA

**FIGURE 3:**
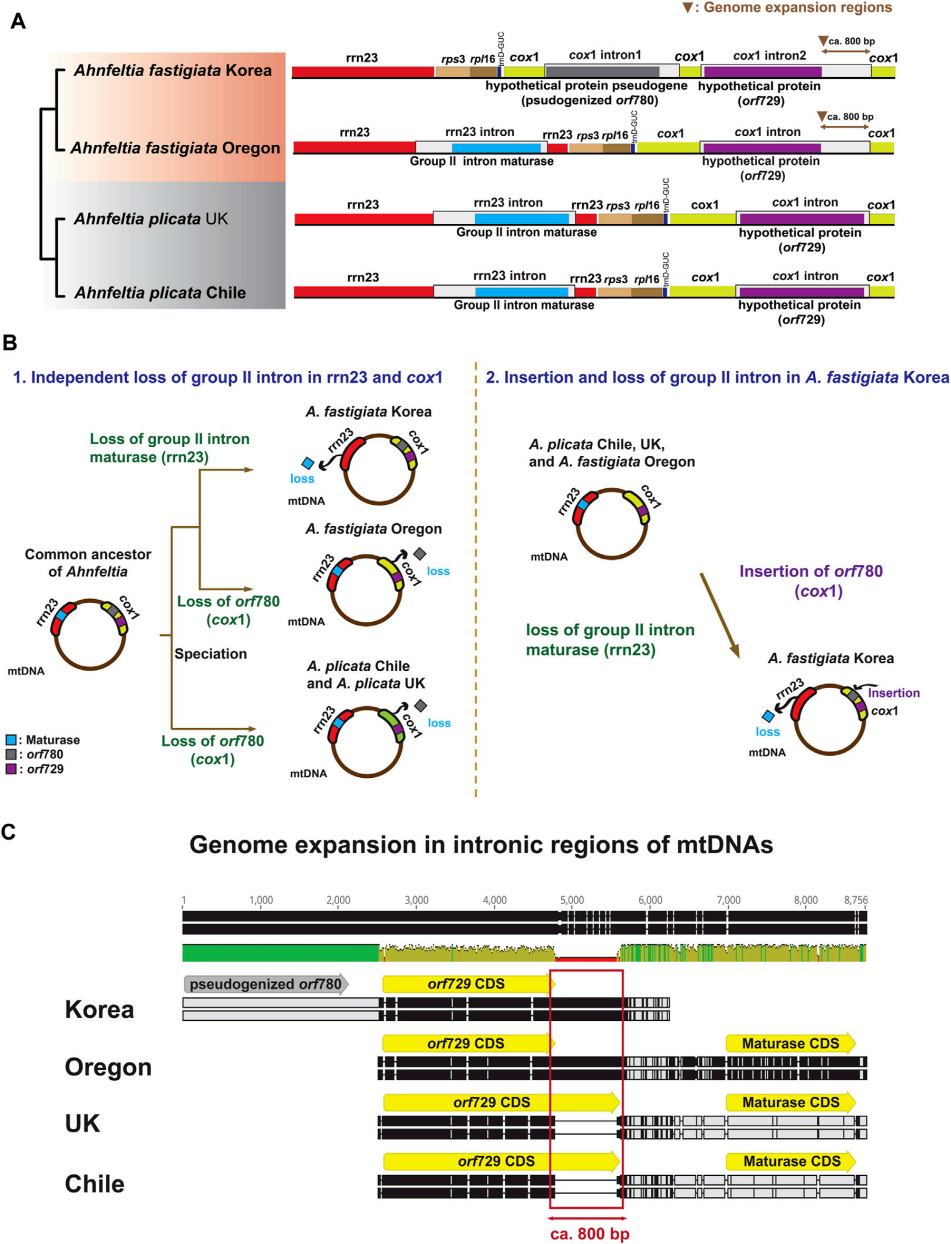
**(A)** OGDRAW plots of group II intronic ORF in mtDNAs of *Ahnfeltia*. A maturase existed in 23S rRNA (rrn23) and *orf729* was present in *cox1* in *A. plicata* Chile, *A. plicata* UK, and *A. fastigiata* Oregon. Two group II intronic ORFs *orf729* (*cox1*) and *orf780* (*cox1*) existed in *cox1* in *A. fastigiata* Korea. Genome expansion occurred in *orf729* (*cox1*) in *A. fastigiata*. **(B)** Two scenarios for different group II intronic ORFs composition in mtDNAs of *Ahnfeltia*. In first scenario, from a common ancestor, three groups might have lost the maturase and *orf780*, independently. In second scenario, *A. fastigiata* Korea might have lost the maturase and gained *orf780*. **(C)** Alignments of intronic regions of mtDNAs showing genome extended regions. About 800 base pairs were different in *orf729s*, which made the mtDNA sizes of *A. fastigiata* larger than those of *A. plicata*.

A distinct difference among the mtDNAs of *Ahnfeltia* was in the group II intronic ORF composition in 23S rRNA (rrn23) and *cox*1. In *A. plicata* Chile, *A. plicata* UK, and *A. fastigiata* Oregon, a group II intronic ORF, a maturase, existed in rrn23, and another group II intronic ORF, *orf*729, was present in *cox*1. In contrast, in *A. fastigiata* Korea, the maturase was absent in rrn23, but two group II intronic ORFs, *orf*729 and *orf*780, existed in *cox*1. Additionally, there was genome expansion in *cox*1 group II intronic ORF (*orf*729) in both *A. fastigiata* Oregon and Korea. The nonsense mutation was found in *orf*780 in *cox*1 of *A. fastigiata* Korea (Figure 3A). To trace the origin of group II intronic ORFs, we conducted a phylogenetic analysis, which revealed that group II intronic ORFs in mtDNAs had three different origins. Maturases showed a close relationship to cyanobacteria, while *orf*729 and *orf*780 were close to the bacteria but in different clades. Furthermore, *orf*780 had a monophyletic relationship with group II intronic ORF of the Bangiophyceae (Supplementary Figure S2).

### Phylogenetic Relationships and *cox*1 Haplotype Network

**FIGURE 4:**
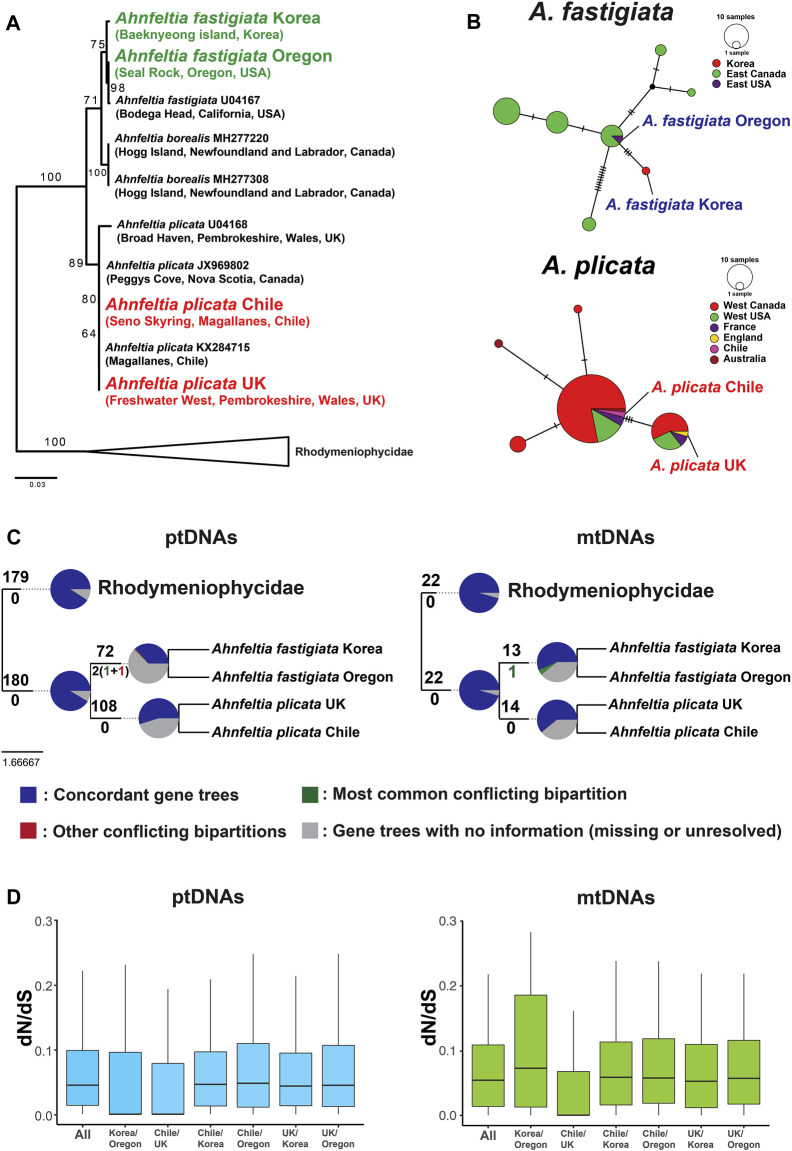
**(A)** *rbc*L phylogeny using currently available *rbc*L sequence of *Ahnfeltia* on NCBI database. Maximum likelihood (ML) bootstrap values (≥50%) are shown at each node. **(B)** *cox*1 haplotype network with COI-5P data of Milstein and Saunders (2012). **(C)** Result of gene tree conflict analysis using ptDNA genes and mtDNA genes of *Ahnfeltia*. In pie charts at each node, the proportion of concordant genes trees within the clade defined by the node is marked in blue and the proportion of discordant gene trees within the clade are marked in green and red. The proportion of main alternatives for the clade are green and the proportion of all other alternatives for the clade are red. The proportion of homologs with less than 80% bootstrap support are grey. **(D)** Boxplots of the average of dN/dS values of ptDNA genes and mtDNA genes. The average of dN/dS of intraspecies appeared to be lower than that of interspecies, but all results were not statistically significant.

Three separate clades (i.e., *A. borealis*, *A. fastigiata*, and *A. plicata*) were supported in the *rbc*L gene phylogeny with the currently available six sequences in the NCBI database. Within the *A. fastigiata* clade, there was distinct *rbc*L divergence between sequences from Korea and Oregon (Figure 4A). The *cox*1 haplotype network also showed that genetic divergence occurred among the four *Ahnfeltia* populations. In the *A. fastigiata* haplotype network, *A. fastigiata* Korea had an independent haplotype segregated from the NE Pacific haplotype groups. In the *A. plicata* haplotype network, *A. plicata* Chile and *A. plicata* UK were in two different haplotype groups (Figure 4B). Phylogenetic trees with concatenated genes from ptDNAs and mtDNAs were congruent with each other and supported currently accepted phylogenetic relationships among the five subclasses in the Florideophyceae (i.e., Hildenbrandiophycidae, Nemaliophycidae, Corallinophycidae, Ahnfeltiophycidae, and Rhodymeniophycidae) (see Supplementary Figures S3, S4). Within the *Ahnfeltia* clade, concatenated gene trees of both ptDNAs and mtDNAs showed a clear separation between *A. plicata* and *A. fastigiata*. To test if the individual gene trees support the concatenated tree, we compared all possible individual gene trees; 197 genes of ptDNA and 23 genes of mtDNA were tested. 180 ptDNA genes and 22 mtDNA genes supported the monophyly of Ahnfeltiophycidae, while 108 and 72 ptDNA genes supported the monophyly of *A. plicata* and *A. fastigiata*, respectively. In addition, 14 and 13 mtDNA genes supported the monophyly of *A. plicata* and *A. fastigiata*, respectively (Figure 4C).

### Non-Synonymous Substitution (dN) and Synonymous Substitution (dS) Ratio

Generally, dN/dS ratios of ptDNA genes were similar to those of mtDNA genes (Figure 4D). dN/dS values from the interspecific comparison were higher than those from the intraspecific comparisons, but they were not statistically significant. dN/dS of most genes from ptDNAs and mtDNAs showed a purifying selection (dN/dS < 1). Several genes had likely undergone positive selection in dN/dS analysis (e.g., *atpG*, *ftr*B, *ntc*A, *psa*D, *psa*E, *psa*I, *rpl*36, etc.). However, in most cases, the outliers likely resulted from biases caused by their short sequence length (e.g., *psa*I, *rpl*36 *rpo*Z, *ycf*33, etc.) and the highly conserved genes within species (e.g., *atp*G, *ntc*A, *psa*D, etc.). In contrast, *psa*E showed a strong positive selection (dN/dS > 1) (Supplementary Table S4). Synonymous substitution (dS) genes occurred more frequently than non-synonymous substitutions (dN) in ptDNAs and mtDNAs of the four *Ahnfeltia* strains (Supplementary Figure S5). Overall dN and dS values of mtDNA genes were higher than those of ptDNA genes, indicating that mtDNA genes have diversified more dynamically than ptDNA genes (Supplementary Figure S5, Supplementary Tables S4, S5). dN and dS of ptDNA genes in *A. fastigiata* were generally higher than those in *A. plicata,* but not statistically significant than those in *A. plicata* (Supplementary Figures S5C, S5E).

## Discussion

### Variations of Organelle Genome Architecture

Our study showed that two events of genome architecture variation happened in *Ahnfeltia*: the translocation of the pseudogenized hypothetical protein region in ptDNA of *A. fastigiata* Oregon, and the different composition of group II intronic ORF in mtDNA of *A. fastigiata* Korea. These genomic variations were distinct features of the organelle genomes of *Ahnfeltia*, but variations in organelle genome structures did not occur between the two *Ahnfeltia* species, *A. fastigiata* and *A. plicata*. This suggested a relatively recent speciation event perhaps 2-5 Mya (Bringloe and Saunders, 2019). In contrast, structures of ptDNAs and mtDNAs in *A. fastigiata* from two different populations (i.e., *A. fastigiata* Korea and Oregon) were different, suggesting different intraspecific selection pressures on different organelles. Based on these results, we postulate that geographic distribution in the North Pacific may have strongly influenced the divergence of organelle genomes of *A. fastigiata*. Several biogeographic studies have revealed high genetic diversity of marine organisms in the North Pacific and have shown a deep genetic separation within amphi-North Pacific taxa (Kai et al., 2011; Yang et al., 2020). In particular, Yang et al. (2020) showed a clear genetic diversity between the NE Pacific and NW Pacific *Gloiopeltis furcata* strains. They also suggested that East Asia in the NW Pacific is a genetic diversity hotspot.

*A. fastigiata* Korea was collected only from the westernmost island of South Korea in the Yellow Sea between the Western Korean peninsula and Eastern China. This island appeared to be acting as a refugium, possibly enabling the divergence of *A. fastigiata* after a marine transgression to the North Yellow Sea after the last glacial maximum in the late Pleistocene. Since *Ahnfeltia* is adapted to colder water (Lüning and Freshwater, 1988; Mansilla et al., 2014), the warm Kuroshio and Yellow Sea currents may have acted as an environmental barrier to isolate the Yellow Sea *A. fastigiata* population (Figure 1B). This geographic isolation may have led to the differentiation of the genomic architecture of *A. fastigiata*. Several studies have reported that marine organisms in the Yellow Sea have distinct genetic diversity compared to organisms in other East Asian regions, perhaps due to the past fluctuation of sea level during glacial periods and present-day geographic barriers including warm currents and freshwater inflow (Yang et al., 2009; Hu et al., 2011; Boo et al., 2019). A phylogeographic study of *Phoca largha* (spotted seals) revealed a distinct genetic signature in populations from the Yellow Sea and Japan (Li et al., 2010). The spotted seal shows an isolated discontinuous distribution similar to that seen in populations of *A. fastigiata* in the northern Yellow Sea and along the NE Pacific coast (Park et al., 2010). To clarify this postulation, further phylogeographic study *on A. fastigiata* is needed in future.

### Putative Translocation Mechanism of ptDNAs

To find out why and how translocation happened in *A. fastigiata* Oregon, we first investigated repeat elements around the translocated regions of four *Ahnfeltia* ptDNAs. Translocation of organelle genomes or organelle genome recombination has frequently happened in many organisms including algae (Kawata et al., 1997; Lin et al., 2015; Choi et al., 2020). Palindromic tandem repeats are thought to function as a hotspot for genome recombination, because they can form hairpin structures following the recombination of complementary sequences (Kawata et al., 1997; Day and Madesis, 2007). Here, palindromic repeats were found to accumulate around translocated regions (Figure 2B). Therefore, it is probable that the translocation of pseudogenized hypothetical protein regions in *A. fastigiata* Oregon was mediated by palindromic repeats.

Another possible explanation for translocated regions of ptDNAs in *A. fastigiata* Oregon relates to red algal plasmids. Plasmids are mobile elements that carry DNA fragments that are involved in organelle genome recombination (Day and Madesis, 2007; Wiedenbeck and Cohan, 2011; Bhattacharya et al., 2018). Lee et al. (2016b) reported several cases of plasmid-mediated horizontal gene transfer, which were found in the organelle genomes of red algae. The hypothetical protein genes in *A. plicata* and the complementary pseudogenized hypothetical protein in *A. fastigiata* are both homologous to the red algal plasmid genes (Figure 2C). Thus, it can be assumed that these hypothetical protein genes were derived from red algal plasmids, followed by pseudogenization in *A. fastigiata*. An interesting point was that intergenic regions of different lengths were found around the hypothetical protein genes and pseudogenes. In *A. plicata* strains, short extra sequences were found around the conserved hypothetical protein genes. In contrast, the hypothetical protein genes in *A. fastigiata* strains had a long extended intergenic region (ca. 3,000–4,000 bp) with a nonsense mutation. This suggests that a hypothetical protein was horizontally introduced by plasmids into the ancestor of the two species; then, the extension of the intergenic region (including homologous tandem repeat copies) in *A. fastigiata* led to the translocation of the hypothetical proteins in *A. fastigiata* Oregon. These sudden plasmid-mediated events are distinguished from conventional cumulative mutations (e.g., haplotype variations of *Ascophyllum nodosum* between the east and west Atlantic, Olsen et al., 2010).

### Gene Gain and Loss Scenarios of Group II Intron in mtDNA

Group II introns, which have been reported in bacteria and organelle genomes, are known to be catalytic ribozymes and mobile elements that enable self-splicing (Lambowitz and Zimmerly, 2004; Zimmerly and Semper, 2015). Group II introns generally include hypothetical proteins (ORF) and maturase in their organelle genomes (Janouškovec et al., 2013; Lee et al., 2016b). Group II intronic ORFs are proliferated in plastid and mitochondrial genomes of green algae and land plants (Del Vasto et al., 2015; Brouard et al., 2016; Repetii et al., 2020) as well as in many red algae (Perrineau et al., 2015; Yang et al., 2015). However, it still remains unclear how intronic ORFs originated and how they degenerate.

In our study, three different group II intronic ORFs were annotated in mtDNAs: *orf*729 and *orf*780 in *cox*1, and maturase in rrn23 (Figure 3A). The phylogenetic analysis revealed that these three group II intronic proteins were independently derived (Supplementary Figure S2). Phylogenetic analysis indicated that *Ahnfeltia* had an independent gene gain or loss in group II intron regions during diversification. Based on these analyses, we suggest two scenarios for intronic ORFs evolution in mtDNAs (Figure 3B).

The first scenario is three independent losses of maturase and *orf*780 (Figure 3B-1). A common ancestor that contained maturase, *orf*729, and *orf*780 (in the group II introns in rrn23 and in *cox*1, respectively) could have lost maturase from rrn23 group II introns of *A. fastigiata* Korea, and then also had a minimum of two independent *orf*780 gene losses in *cox*1 (i.e., one in *A. fastigiata* Oregon, the other in the ancestor of *A. plicata*). Most group II introns in organelle genomes have lost their self-splicing ability because of the accumulation of nonsense transcripts (Zimmerly and Semper, 2015; Bateman, 2020). Thus, if settled group II introns had lost their self-splicing ability in the common ancestor of *Ahnfeltia,* it is more plausible that the redundant group II introns gene loss happened through a differently operated DNA repair system rather than acquisition of introns from external sources.

The second scenario is one gain and one loss in *A. fastigiata* Korea (Figure 3B-2). In this scenario, we can speculate that *A. fastigiata* Korea independently gained *orf*780 in *cox*1 and lost maturase in rrn23, while the other *Ahnfeltia* population retained maturase in rrn23. This scenario is a common explanation based on the mobility of the group II intron (Lambowitz and Zimmerly, 2004; Doolittle, 2014; Zimmerly and Semper, 2015). Because the phylogeny of the intronic ORFs showed that *orf*780 was grouped with *Bangia* and *Pyropia* species (Bangiophyceae) (Supplementary Figure S4), gene transfer could have occurred from epiphytic Bangiophycean species living on the surface of other red algae. This second scenario of two evolutionary events is more parsimonious than the first scenario of three evolutionary events.

### Organelle Genome Expansion and Variation

Assembled organelle genomes of *Ahnfeltia* revealed that genome sizes of both ptDNAs and mtDNAs in *A. fastigiata* are a bit larger than those in *A. plicata*. One clear point is that the genome size difference of *Ahnfeltia* is due to lengths of intergenic regions of the ptDNAs and intronic regions of the mtDNAs (Supplementary Table S2). For the first time, we investigated tandem repeat elements because repeat elements are often considered the main factor for genome expansion (Del Vasto et al., 2015; Repetii et al., 2020; Smith, 2020). Even though they contribute to overall genome expansion, tandem repeats themselves cannot solely explain the genome size difference between the two species (Supplementary Figure S6). We also aligned intergenic and intronic regions of the genomes. The alignment of ptDNAs clearly showed that pseudogenized hypothetical protein regions derived from red algal plasmids in *A. fastigiata* were ca. 3,000–4,000 bp larger than homologous regions in *A. plicata* (Figure 2D). Moreover, the alignment of mtDNAs revealed that the sequence of *cox*1 group II intronic ORF (*orf*729) of *A. fastigiata* remained ca. 800 bp longer than that of *A. plicata* (Figure 3C). Thus, these differences resulted in genome size variation between two species.

Organelle genome expansion in non-coding regions (i.e., intronic and intergenic regions) has been reported from diverse algal lineages (Smith et al., 2010; Del Vasto et al., 2015; Repetii et al., 2020). Organelle genome variation and expansion in non-coding regions have been explained by neutral mutation and random genetic drift rather than natural selection (Kimura, 1968; Smith, 2016). In particular, the mutational hazard hypothesis (MHH) suggests that excess DNA can be a mutational liability that accounts for the accumulation of hazardous DNA sequence in genomes of organisms with a low mutation rate (μ) and small effective population size (N_e_) (Lynch et al., 2006; Smith, 2016). Moreover, (Christensen, 2013; Christensen, 2014) suggests that different DNA repair mechanisms function between coding regions and neutral mutated non-coding regions after double-strand break DNA, which can induce genomic expansion and rearrangement. Therefore, organelle genome differences in *Ahnfeltia* might have occurred since the neutral mutation (following the MHH hypothesis) had occurred in sequences that originated externally in non-coding regions. Then the repair system operated differently in each taxon when they speciated and diversified. The finding of plasmid-derived hypothetical protein pseudogenes in ptDNAs and pseudogenized group II intronic-derived ORF (*orf*780) in mtDNAs of *A. fastigiata* also indicates that the exotic genes will be degraded in organelle genomes.

### Genetic Divergence of Coding Genes

The coding gene contents of *Ahnfeltia* mtDNAs and ptDNAs were relatively conserved compared to their genome architecture variation. In dN/dS analysis, most coding genes were under purifying selection as synonymous substitution (dS) rates were higher than those of non-synonymous substitution (dN) (Supplementary Figure S5). One case, *psa*E, appeared to be under positive selection with a high dN/dS ratio (1.253) (Table S2). *psa*E is a short gene coding for one of the subunits of the photosystem I complex (PSI), consisting of 62 amino acids. PsaE subunit joins the docking of ferredoxin and flavodoxin on the stromal side of the membrane (Fromme et al., 2003). It has a strong interaction with several subunits including PsaA and PsaB, suggesting that this interaction is important for the interaction of PSI with the phycobilisomes (Fromme et al., 2003; Vanselow et al., 2009). Therefore, the positive selection of the *psa*E gene suggests differentiation of photosynthetic ability in different populations. In red algae, the *psa*E gene is in the plastid genome (Lee et al., 2016a), while it is located in the nuclear genome of land plants (Rivas et al., 2002; Wicke et al., 2011; Moghaddam and Kazempour-Osaloo, 2020), suggesting a differential evolution of PSI subunits in photosynthetic organisms (Rivas et al., 2002).

Although gene contents were conserved in organelle genomes, there was genetic diversity between different biogeographic regions. The *rbc*L gene phylogeny and *cox*1 haplotype network indicated that regional genetic diversification had occurred in the four *Ahnfeltia* populations (Figures 4A,B). In the *cox*1 haplotype network (including all published *Ahnfeltia cox*1 sequence data), *A. plicata* Chile and UK belonged to two different haplotypes separated by three haplotype steps, and *A. fastigiata* Korea and Oregon showed a similar genetic distance between two different haplotypes. Tree topology analysis using individual genes also indicated that Ahnfeltiophycidae is highly conserved and most of the ptDNAs and mtDNAs genes supported this subclass as an independent lineage. Around half of ptDNA genes and mtDNA genes supported the topology of this tree with >80% bootstrap supports, although some genes contradicted the relationship (especially in the two populations of *A. fastigiata*) (Figure 4C).

## Conclusion

Our primary genomic study of *Ahnfeltia* clearly demonstrates that *Ahnfeltia* organelle genomes have been dynamically changing, and genetic divergence of *Ahnfeltia* has enabled it to adapt to factors encountered during its global expansion. Widely separated populations of both *A. fastigiata* and *A. plicata* showed numerous genetic adaptations. The amphi-North Pacific species *A. fastigiata* showed more genomic divergence than the bipolar species, *A. plicata*. In *A. fastigiata*, genome structure variations were present. In the Oregon population, tandem repeats and a plasmid-mediated translocation event in ptDNA had occurred; in the Korean population, a different group II intron ORFs composition in mtDNA was present. On the other hand, stable genomic structures were shown in two *A. plicata* populations: Chile and UK. Overall, our data suggests that geographic distribution has affected the genomic and genetic characteristics of *Ahnfeltia.* To further confirm our geographic theory, a future whole-genomic study at the population-level is needed that incorporates more extensive worldwide sampling along with comparisons of both historic and present day geological and ecological data in each area.

## Data Availability

The datasets presented in this study can be found in online repositories. The names of the repository/repositories and accession number(s) can be found below: Newly sequenced ptDNAs of *A. plicata* Chile (MZ393453), *A. plicata* UK (MZ393452), *A. fastigiata* Korea (MZ393455) and *A. fastigiata* Oregon (MZ393454), and mtDNAs of *A. plicata* Chile (MZ393458), *A. plicata* UK (MZ393459), *A. fastigiata* Korea (MZ393456) and *A. fastigiata* Oregon (MZ393457) have been deposited in the NCBI GenBank database.
