# Supplementary material for: Organelle Genome Variation in the Red Algal Genus Ahnfeltia (Florideophyceae)
Source: Front Genet. 2021 Sep 27;12:724734. doi: 10.3389/fgene.2021.724734 (PMC8503264; doi:10.3389/fgene.2021.724734)
Supplement: Supplementary file 1 [file DataSheet1.pdf]

Supplementary Information

for

Organelle genome variation in the red algal genus *Ahnfeltia* (Florideophyceae)

Hocheol Kim[1], Ji Hyun Yang[1], Danilo E. Bustamante[2], Martha S. Calderon[3], Andres Mansilla[3], Christine A. Maggs[4], Gayle I. Hansen[5], and Hwan Su Yoon[1*]

[1]Department of Biological Sciences, Sungkyunkwan University, Suwon 16419, Korea

[2]Instituto de Investigación para el Desarrollo Sustentable de Ceja de Selva (INDES-CES), Universidad Nacional Toribio Rodríguez de Mendoza, Amazonas, Peru

[3]Laboratorio de Macroalgas Antárticas y Subantárticas, Universidad de Magallanes, Casilla 113-D, Punta Arenas, Chile

[4]School of Biological Sciences, Queen's University Belfast BT9 5AJ, UK

[5]Oregon State University, 637 SW 9th Street, #F, Newport, OR 97365, USA

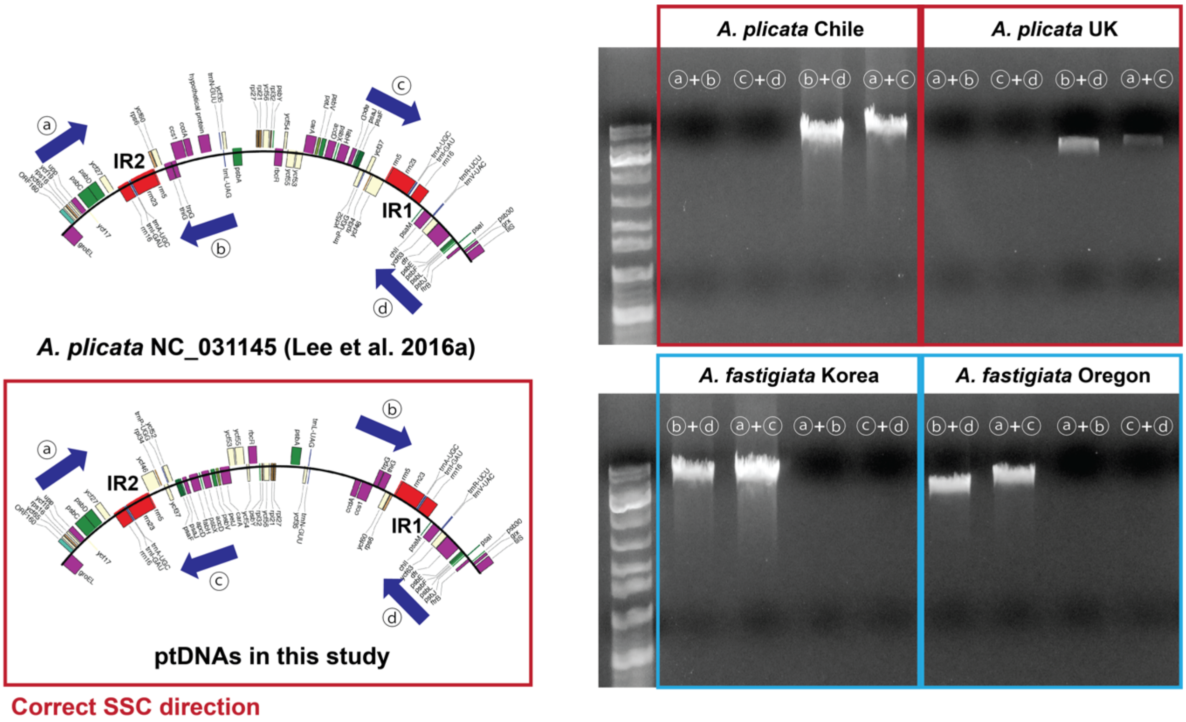

Figure S1. PCR result for the confirmation of direction of small single copy (SSC) regions. For correction direction of SSC to IR1, *rps*6 gene (b) should face to *psa*M and *Chl*I (d). For correct direction of SSC to IR2, *ycf*27 (a) should confront to *ycf*46 (c).

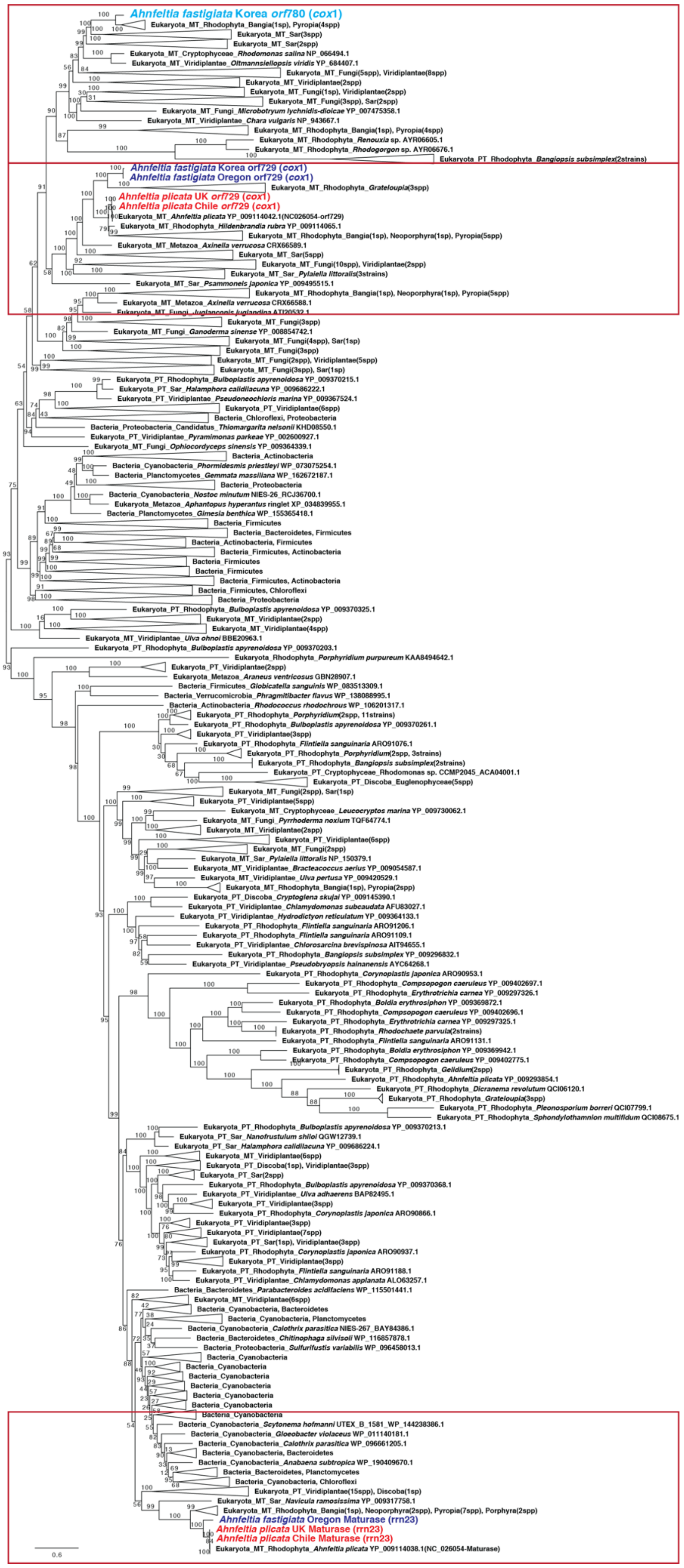

Figure S2. The phylogenetic tree of group II intron ORFs in mtDNAs of *Ahnfeltia.*

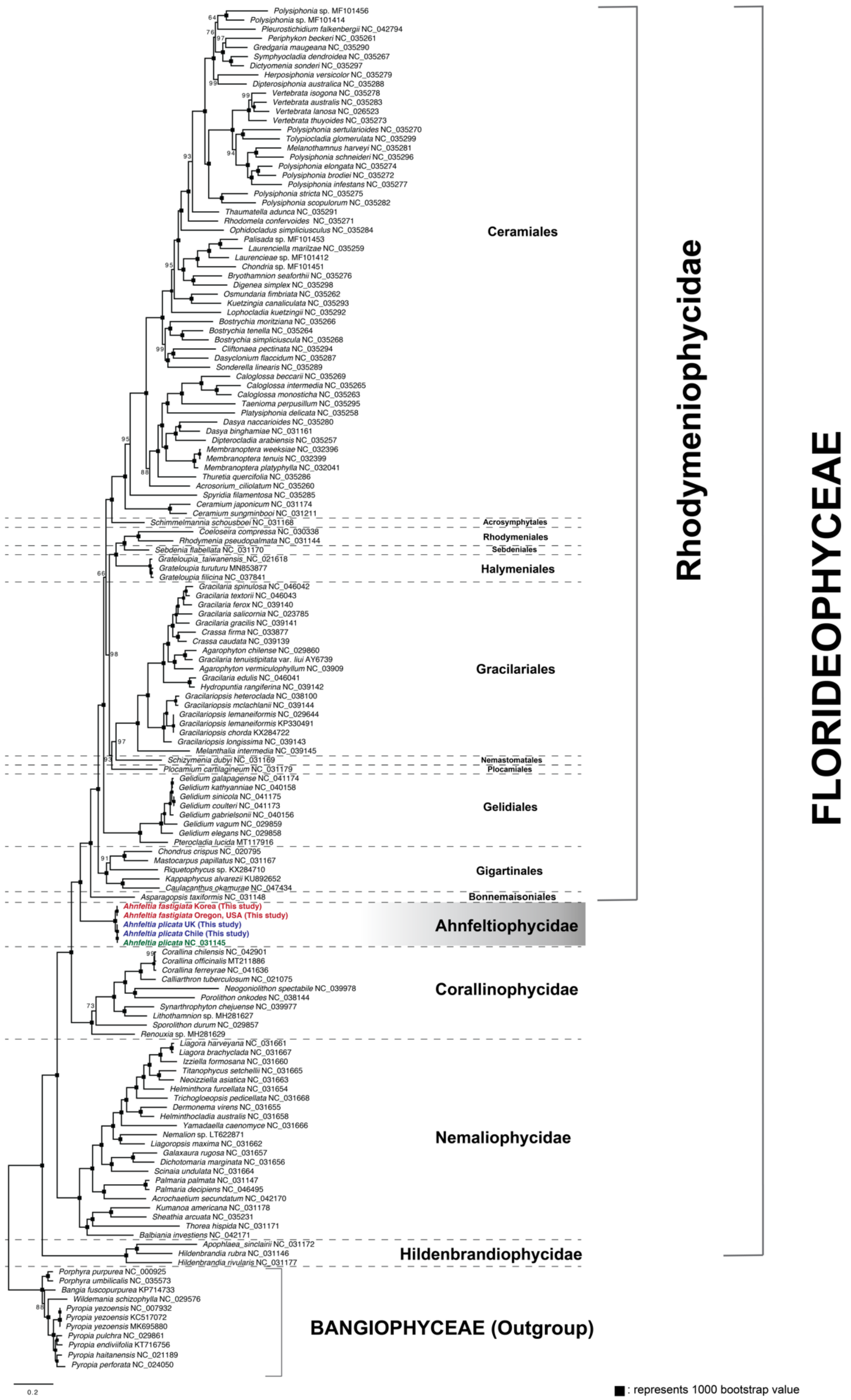

Figure S3. Phylogeny of red algae using 130 concatenated genes from 149 ptDNAs of red algae. Maximum likelihood method was used to construct tree. Dark rectangle marks on each node represent 1000 bootstrap supporting value.

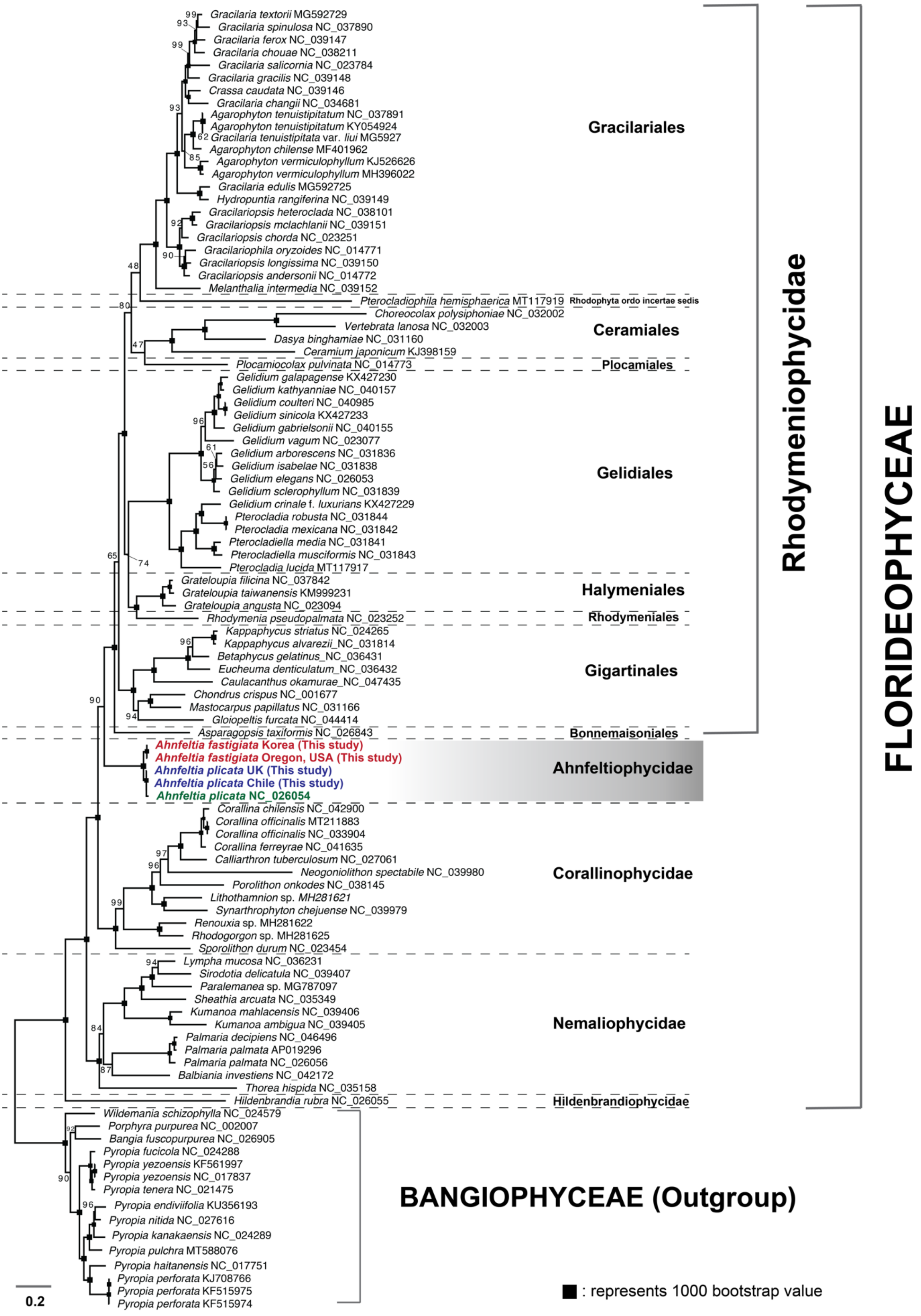

Figure S4. Phylogeny of red algae using 11 concatenated genes from 103 mtDNAs of red algae. Maximum likelihood method was used to construct tree. Dark rectangle marks on each node represent 1000 bootstrap supporting value.

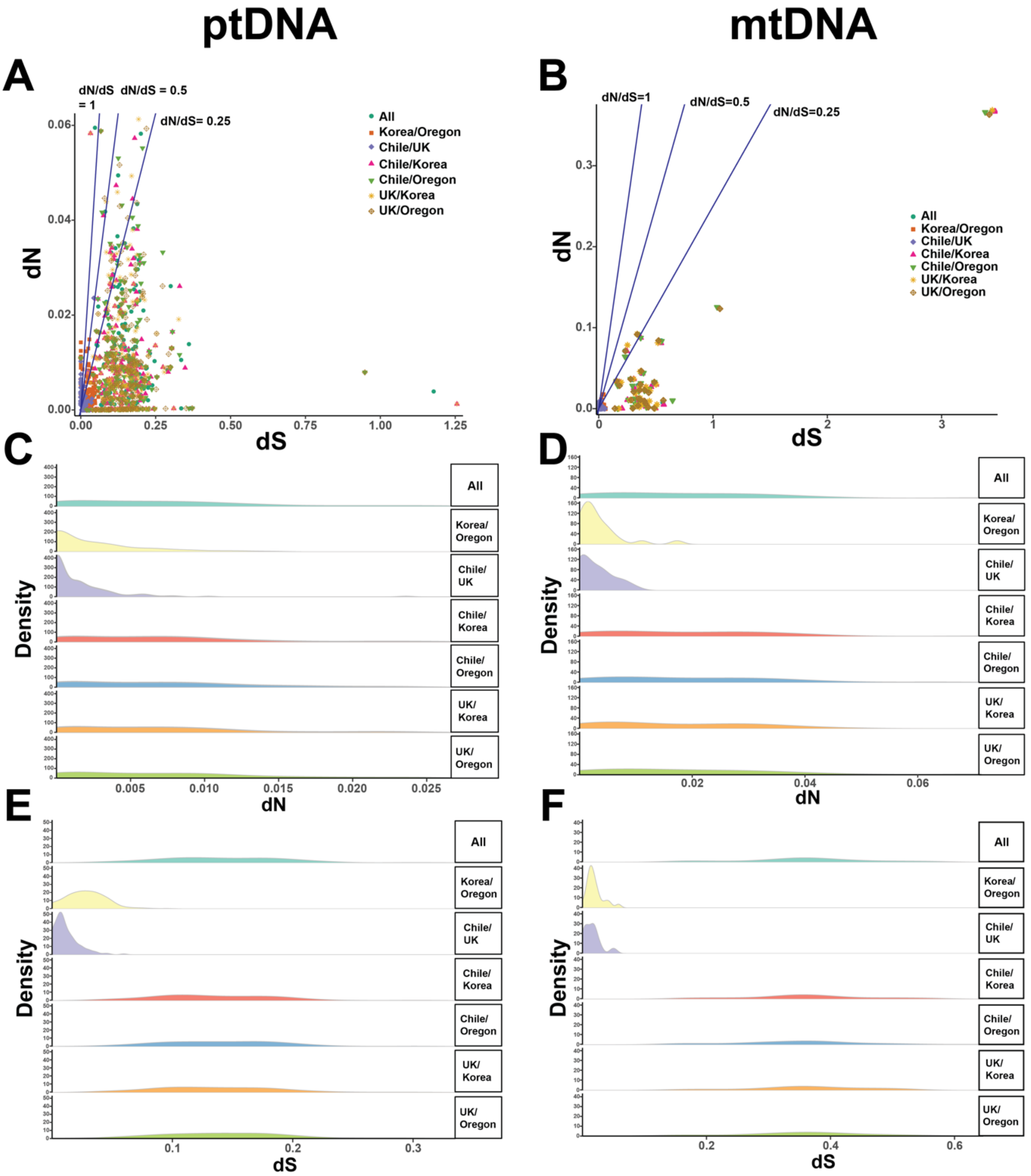

Figure S5. Results of nonsynonymous substitution (dN) and synonymous substitution (dS) analysis. (A) Dot plot of dN/dS ratio of ptDNA genes. (B) Dot plot of dN/dS ratio of mtDNA genes. (C) Density plot of dN of ptDNA genes. (D) Density plot of dN of mtDNA genes. (E) Density plot of dS of ptDNA genes. (F) Density plot of dS of mtDNA. genes.

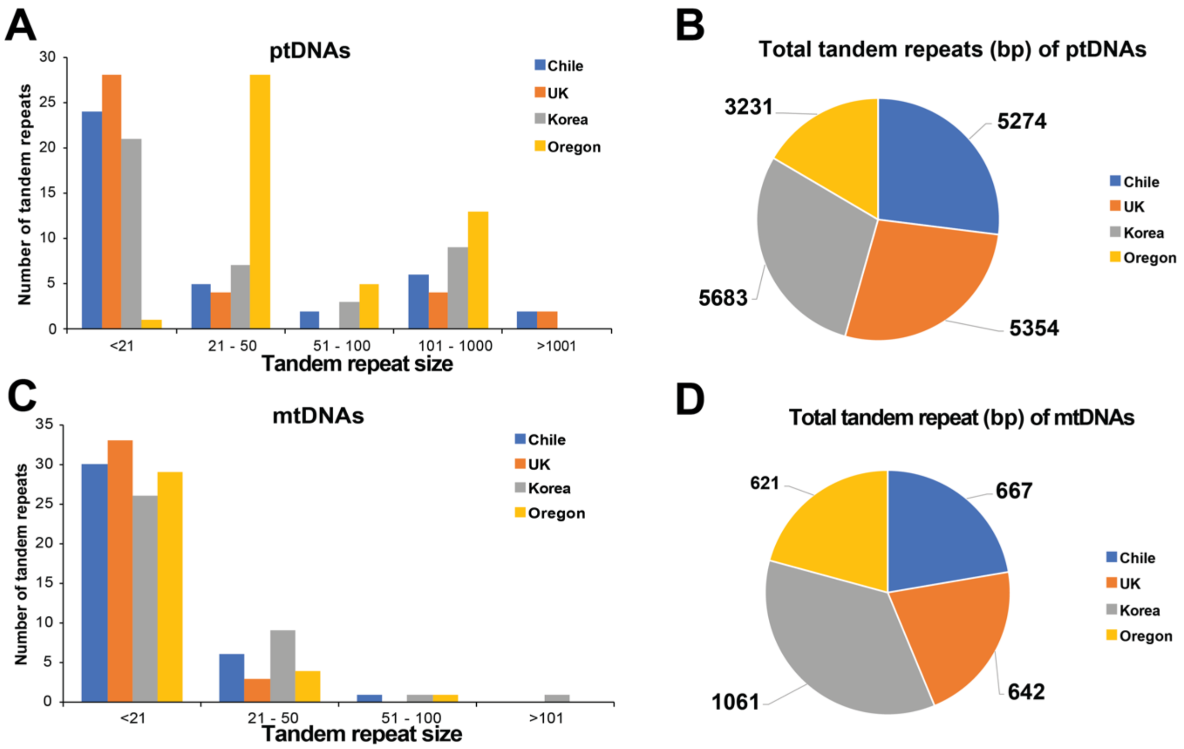

Figure S6. Overview of tandem repeat elements in organelle genomes of *Ahnfeltia*. (A) Number of tandem repeats and its frequency in each ptDNA. (B) Total tandem repeats of each ptDNA. (C) Number of tandem repeats and its frequency in each mtDNA. (D) Total tandem repeats of each mtDNA.

Table S1. Species and GeneBank Accession information used in *rbc*L gene phylogeny.

Species	GeneBank Accession	Reference
Ahnfeltia borealis	MH277220	-
Ahnfeltia borealis	MH277308	(Bringloe and Saunders, 2019)
Ahnfeltia fastigiata	U04167	(Freshwater et al., 1994)
Ahnfeltia plicata	JX969802	(Dixon and Saunders, 2013)
Ahnfeltia plicata	KX284715	(Lee et al., 2016a)
Ahnfeltia plicata	U04168	(Freshwater et al., 1994)

Table S2. PCR primer information for SSC confirmation of *Ahnfeltia.*

	**Direction**	**Primer name**	**Sequence (5′ to 3′)**
A. plicata	ⓐ	Fas_ycf27	TGTATCCACTTTTAAGAAGCCT
	ⓑ	Pli_rps6	GAGCCCTAGTTCAGATTTTAGA
	ⓒ	Pli_rpl34	GAATGAAAACAGTCAGTGGTAG
	ⓓ	Pli_ChlI	AGAATACCCCTGTTAGCTTTAG
A. fastigiata	ⓐ	Fas_ycf27	TGTATCCACTTTTAAGAAGCCT
	ⓑ	Fas_ycf46	TGTACGCATGATGATAAGGTTA
	ⓒ	Fas_rps6	TGGAGCCTTAGTTCAGATTTTA
	ⓓ	Fas_ChlI	CCTGTTTCTTCCCTTTATATGC

Table S3. Summary of DNA sequence length of ptDNAs and mtDNAs of *Ahnfeltia*.

	**ptDNAs**				**mtDNAs**			
	A. fastigiata **Korea**	***A. fastigiata*** **Oregon**	***A. plicata*** **UK**	***A. plicata*** **Chile**	***A. fastigiata*** **Korea**	***A. fastigiata*** **Oregon**	***A. plicata*** **UK**	***A. plicata*** **Chile**
**Coding region**	160,034	158,620	161,197	161,206	28,009	27,552	27,594	27,567
**Intergenic region**	33,584	34,048	29,866	29,832	3,581	3,423	3,706	3,783
**Intronic region**	2,022	2,022	2,022	2,022	6,198	6,171	5,390	5,362

Table S4. Result of dN/dS analysis of ptDNA genes.

	All	Korea /Oregon	Chile /UK	Chile /Korea	Chile /Oregon	UK /Korea	UK /Oregon
**ORF160**	0.158	0.297	NA	0.157	0.194	0.157	0.194
**ORF426**	0.396	0.348	47.861	0.401	0.423	0.375	0.399
**ORF64**	0.111	0.001	0.355	0.083	0.079	0.156	0.147
**ORF745**	0.327	0.179	0.512	0.339	0.357	0.319	0.337
**accA**	0.081	0.061	0.114	0.067	0.073	0.090	0.065
**accB**	0.244	0.337	NA	0.228	0.263	0.228	0.263
**accD**	0.036	0.187	NA	0.047	0.021	0.047	0.021
**acpP**	0.023	0.001	0.001	0.027	0.030	0.027	0.030
**acsF**	0.132	0.001	0.198	0.136	0.138	0.132	0.134
**apcA**	0.001	NA	NA	0.001	0.001	0.001	0.001
**apcB**	0.001	0.001	0.001	0.001	0.001	0.001	0.001
**apcD**	0.128	0.079	NA	0.114	0.122	0.114	0.122
**apcE**	0.065	0.064	0.071	0.065	0.074	0.059	0.067
**apcF**	0.040	0.073	0.443	0.036	0.055	0.034	0.051
**argB**	0.034	0.132	0.180	0.044	0.032	0.036	0.024
**atpA**	0.015	0.032	0.001	0.019	0.011	0.020	0.011
**atpB**	0.025	0.001	0.194	0.026	0.022	0.033	0.028
**atpD**	0.267	0.767	0.261	0.245	0.281	0.251	0.281
**atpE**	0.066	0.212	NA	0.067	0.078	0.067	0.078
**atpF**	0.055	0.283	NA	0.063	0.045	0.063	0.045
**atpG**	0.057	0.291	50	0.085	0.058	0.054	0.027
**atpH**	0.001	0.001	0.001	0.001	0.001	0.001	0.001
**atpI**	0.009	0.522	NA	0.018	0.001	0.018	0.001
**bas1**	0.054	0.001	0.001	0.056	0.058	0.051	0.053
**carA**	0.142	0.091	0.001	0.164	0.126	0.160	0.124
**cbbX**	0.013	0.001	0.188	0.013	0.012	0.015	0.014
**ccdA**	0.299	0.519	0.001	0.288	0.314	0.306	0.330
**ccs1**	0.072	0.071	0.001	0.064	0.080	0.067	0.084
**ccsA**	0.051	0.344	0.001	0.049	0.059	0.046	0.055
**cemA**	0.022	0.035	0.001	0.030	0.018	0.028	0.017
**chlI**	0.027	0.001	NA	0.027	0.027	0.027	0.027
**clpC**	0.003	0.025	0.001	0.003	0.003	0.004	0.003
**cpcA**	0.009	0.001	0.001	0.010	0.009	0.012	0.011
**cpcB**	0.009	0.088	NA	0.001	0.018	0.001	0.018
**cpcG**	0.046	0.042	0.045	0.060	0.052	0.041	0.035
**cpcS**	0.239	0.517	0.001	0.227	0.272	0.212	0.250
**cpeA**	0.222	0.001	NA	0.328	0.181	0.328	0.181
**cpeB**	0.016	NA	0.063	0.001	0.001	0.029	0.029
**dfr**	0.030	0.001	0.785	0.035	0.035	0.026	0.026
**dnaB**	0.259	0.041	0.182	0.291	0.248	0.273	0.233
**dnaK**	0.045	0.001	0.001	0.046	0.043	0.049	0.045
**fabH**	0.036	0.001	NA	0.035	0.036	0.035	0.036
**ftrB**	0.020	0.095	50	0.016	0.041	0.001	0.022
**ftsH**	0.016	0.031	0.001	0.020	0.013	0.021	0.013
**gltB**	0.047	0.098	0.140	0.044	0.050	0.044	0.049
**groEL**	0.012	0.001	0.001	0.013	0.014	0.013	0.013
**grx**	0.037	0.001	NA	0.030	0.042	0.030	0.042
**hypothetical protein**	NA	NA	0.543	NA	NA	NA	NA
**ilvB**	0.029	0.114	0.001	0.027	0.029	0.029	0.031
**ilvH**	0.017	0.001	NA	0.018	0.017	0.018	0.017
**infB**	0.127	0.112	0.551	0.134	0.133	0.121	0.121
**infC**	0.044	0.001	NA	0.042	0.046	0.042	0.046

	All	Korea /Oregon	Chile /UK	Chile /Korea	Chile /Oregon	UK /Korea	UK /Oregon
**moeB**	0.123	0.105	0.001	0.152	0.135	0.139	0.125
**nblA**	0.137	0.313	NA	0.150	0.123	0.150	0.123
**ntcA**	0.092	0.084	49.756	0.073	0.102	0.087	0.119
**odpA**	0.026	0.118	NA	0.025	0.030	0.025	0.030
**odpB**	0.008	0.001	0.001	0.008	0.009	0.008	0.011
**orf83**	0.230	50	50	0.257	0.203	0.291	0.233
**pbsA**	0.056	0.001	0.001	0.059	0.054	0.056	0.052
**petA**	0.035	0.070	0.091	0.044	0.036	0.034	0.027
**petB**	0.014	0.091	NA	0.026	0.001	0.026	0.001
**petD**	0.030	0.001	0.001	0.047	0.035	0.034	0.026
**petF**	0.039	NA	NA	0.041	0.041	0.041	0.041
**petG**	0.001	NA	NA	0.001	0.001	0.001	0.001
**petJ**	0.123	0.190	NA	0.114	0.128	0.114	0.128
**petL**	0.001	NA	NA	0.001	0.001	0.001	0.001
**petM**	0.001	0.001	NA	0.001	0.001	0.001	0.001
**petN**	0.001	NA	NA	0.001	0.001	0.001	0.001
**petP**	0.043	NA	NA	0.043	0.043	0.043	0.043
**pgmA**	0.074	0.048	0.170	0.080	0.077	0.074	0.071
**preA**	0.024	0.001	0.405	0.032	0.042	0.011	0.015
**psaA**	0.009	0.001	0.001	0.010	0.009	0.010	0.009
**psaB**	0.016	0.015	0.001	0.016	0.017	0.015	0.016
**psaC**	0.110	NA	NA	0.110	0.110	0.110	0.110
**psaD**	0.120	0.001	50	0.173	0.179	0.101	0.103
**psaE**	**1.253**	0.001	NA	**1.794**	**0.878**	**1.794**	**0.878**
**psaF**	0.099	0.244	0.001	0.121	0.090	0.109	0.081
**psaI**	0.122	50	NA	0.100	0.178	0.100	0.178
**psaJ**	0.001	0.001	NA	0.001	0.001	0.001	0.001
**psaK**	0.044	NA	NA	0.049	0.049	0.049	0.049
**psaL**	0.001	0.001	0.001	0.001	0.001	0.001	0.001
**psaM**	NA	NA	NA	NA	NA	NA	NA
**psb30**	0.112	NA	NA	0.111	0.111	0.111	0.111
**psbA**	0.001	0.001	0.001	0.001	0.001	0.001	0.001
**psbB**	0.006	0.001	0.123	0.010	0.009	0.005	0.004
**psbC**	0.004	0.001	0.001	0.005	0.004	0.005	0.004
**psbD**	0.001	0.001	0.001	0.001	0.001	0.001	0.001
**psbE**	0.001	0.001	NA	0.001	0.001	0.001	0.001
**psbF**	0.025	NA	50	0.001	0.001	0.056	0.056
**psbH**	0.001	NA	NA	0.001	0.001	0.001	0.001
**psbI**	0.099	0.001	NA	0.096	0.164	0.096	0.164
**psbJ**	0.001	NA	0.001	0.001	0.001	0.001	0.001
**psbK**	0.001	0.001	NA	0.001	0.001	0.001	0.001
**psbL**	0.001	NA	NA	0.001	0.001	0.001	0.001
**psbN**	0.090	50	0.001	0.151	0.073	0.091	0.045
**psbT**	0.001	NA	0.001	NA	NA	0.001	0.001
**psbV**	0.050	0.001	50	0.072	0.062	0.036	0.031
**psbW**	0.001	NA	0.001	0.001	0.001	0.001	0.001
**psbX**	0.001	NA	NA	0.001	0.001	0.001	0.001
**psbY**	NA	NA	NA	NA	NA	NA	NA
**psbZ**	0.001	NA	NA	0.001	0.001	0.001	0.001
**rbcL**	0.027	0.190	0.001	0.037	0.023	0.036	0.022
**rbcR**	0.001	0.001	0.001	0.001	0.001	0.001	0.001
**rbcS**	0.055	0.001	0.001	0.067	0.066	0.048	0.049
**rne**	0.057	0.093	0.232	0.056	0.063	0.050	0.057
**rpl11**	0.001	0.001	0.001	0.001	0.001	0.001	0.001

	All	Korea /Oregon	Chile /UK	Chile /Korea	Chile /Oregon	UK /Korea	UK /Oregon
**rpl12**	0.095	0.001	NA	0.094	0.116	0.094	0.116
**rpl13**	0.192	0.204	0.001	0.209	0.210	0.179	0.183
**rpl14**	0.001	0.001	0.001	0.001	0.001	0.001	0.001
**rpl16**	0.149	0.001	0.190	0.182	0.137	0.175	0.119
**rpl18**	0.119	0.137	50	0.261	0.133	0.190	0.098
**rpl19**	0.078	0.001	NA	0.073	0.087	0.073	0.087
**rpl1**	0.067	0.041	0.664	0.045	0.058	0.073	0.085
**rpl20**	0.058	50	50	0.001	0.036	0.077	0.113
**rpl21**	0.060	0.001	NA	0.066	0.052	0.066	0.052
**rpl22**	0.095	0.001	0.001	0.121	0.090	0.121	0.090
**rpl23**	0.235	0.001	0.001	0.273	0.184	0.275	0.186
**rpl24**	0.145	50	NA	0.158	0.156	0.158	0.156
**rpl27**	0.021	0.071	NA	0.017	0.050	0.017	0.050
**rpl28**	0.065	0.001	NA	0.061	0.067	0.061	0.067
**rpl29**	0.074	0.197	50	0.074	0.151	0.001	0.071
**rpl2**	0.054	0.029	0.439	0.056	0.048	0.063	0.055
**rpl31**	0.042	50	NA	0.054	0.027	0.054	0.027
**rpl32**	0.003	0.163	NA	0.001	0.008	0.001	0.008
**rpl33**	0.088	50	NA	0.068	0.123	0.068	0.123
**rpl34**	0.040	0.001	NA	0.051	0.042	0.051	0.042
**rpl35**	0.143	0.001	50	0.183	0.160	0.131	0.116
**rpl36**	50	50	NA	NA	50	NA	50
**rpl3**	0.071	0.001	0.001	0.072	0.078	0.067	0.072
**rpl4**	0.061	0.098	NA	0.069	0.054	0.069	0.054
**rpl5**	0.048	0.068	NA	0.040	0.057	0.040	0.057
**rpl6**	0.204	0.147	0.001	0.270	0.152	0.305	0.167
**rpl9**	0.374	NA	50	0.407	0.407	0.348	0.348
**rpoA**	0.013	0.053	50	0.015	0.026	0.001	0.013
**rpoB**	0.029	0.001	0.097	0.029	0.026	0.033	0.030
**rpoC1**	0.017	0.070	0.001	0.025	0.012	0.024	0.012
**rpoC2**	0.044	0.036	0.090	0.043	0.052	0.038	0.046
**rpoZ**	0.260	42.889	NA	0.203	0.317	0.203	0.317
**rps10**	0.001	0.001	0.001	0.001	0.001	0.001	0.001
**rps11**	0.001	NA	NA	0.001	0.001	0.001	0.001
**rps12**	0.001	0.001	NA	0.001	0.001	0.001	0.001
**rps13**	0.012	50	NA	0.024	0.001	0.024	0.001
**rps14**	0.062	0.353	NA	0.032	0.082	0.032	0.082
**rps16**	0.052	0.001	NA	0.052	0.067	0.052	0.067
**rps17**	0.038	NA	0.001	0.054	0.054	0.064	0.064
**rps18**	0.001	0.001	NA	0.001	0.001	0.001	0.001
**rps19**	0.083	50	NA	0.083	0.083	0.083	0.083
**rps1**	0.030	0.079	0.001	0.033	0.030	0.033	0.030
**rps20**	0.073	50	0.001	0.049	0.097	0.053	0.105
**rps2**	0.035	0.001	0.115	0.038	0.041	0.029	0.031
**rps3**	0.027	0.047	0.001	0.030	0.027	0.033	0.030
**rps4**	0.059	0.142	NA	0.065	0.053	0.065	0.053
**rps5**	0.010	0.001	0.264	0.001	0.001	0.025	0.019
**rps6**	0.001	0.001	NA	0.001	0.001	0.001	0.001
**rps7**	0.019	0.001	NA	0.027	0.024	0.027	0.024
**rps8**	0.017	0.001	NA	0.021	0.017	0.021	0.017
**rps9**	0.001	0.001	NA	0.001	0.001	0.001	0.001
**secA**	0.052	0.129	0.052	0.055	0.054	0.051	0.050
**secG**	0.094	NA	NA	0.093	0.093	0.093	0.093
**secY**	0.050	0.072	50	0.048	0.078	0.034	0.061

	All	Korea /Oregon	Chile /UK	Chile /Korea	Chile /Oregon	UK /Korea	UK /Oregon
**sufB**	0.046	0.083	0.137	0.047	0.050	0.042	0.044
**sufC**	0.061	0.042	NA	0.059	0.062	0.059	0.062
**syfB**	0.163	0.252	0.700	0.182	0.156	0.175	0.156
**syh**	0.053	0.215	0.322	0.053	0.063	0.047	0.058
**tatC**	0.046	0.001	0.001	0.049	0.043	0.053	0.046
**thiG**	0.021	0.047	50	0.014	0.023	0.020	0.029
**thiS**	0.294	0.001	0.001	0.329	0.222	0.388	0.248
**tilS**	0.173	0.230	NA	0.192	0.165	0.192	0.165
**trpA**	0.031	0.124	0.001	0.025	0.035	0.030	0.040
**trpG**	0.100	0.187	0.001	0.123	0.096	0.109	0.086
**trxA**	0.001	0.001	NA	0.001	0.001	0.001	0.001
**tsf**	0.130	0.001	49.031	0.197	0.142	0.154	0.114
**tufA**	0.001	0.001	0.001	0.001	0.001	0.001	0.001
**upp**	0.156	1.422	0.079	0.157	0.147	0.170	0.156
**ycf17**	0.001	0.001	NA	0.001	0.001	0.001	0.001
**ycf19**	0.001	0.001	0.001	0.001	0.001	0.001	0.001
**ycf20**	0.100	0.085	NA	0.103	0.114	0.103	0.114
**ycf21**	0.512	0.001	0.343	0.539	0.586	0.517	0.560
**ycf22**	0.178	0.001	0.222	0.198	0.168	0.200	0.168
**ycf23**	0.184	0.295	0.112	0.230	0.165	0.214	0.157
**ycf27**	0.001	0.001	NA	0.001	0.001	0.001	0.001
**ycf29**	0.094	0.168	50	0.073	0.107	0.092	0.130
**ycf33**	0.071	50	NA	0.128	0.001	0.128	0.001
**ycf34**	0.087	0.397	50	0.079	0.121	0.058	0.096
**ycf35**	0.143	0.231	NA	0.124	0.160	0.124	0.160
**ycf36**	0.176	0.001	NA	0.190	0.174	0.190	0.174
**ycf37**	0.218	0.077	0.001	0.202	0.235	0.219	0.257
**ycf38**	0.072	0.001	0.001	0.076	0.075	0.074	0.073
**ycf39**	0.128	0.164	0.267	0.151	0.102	0.155	0.107
**ycf3**	0.034	0.001	0.001	0.042	0.030	0.045	0.032
**ycf41**	0.209	0.001	0.001	0.262	0.205	0.234	0.187
**ycf45**	0.064	0.106	0.001	0.066	0.062	0.064	0.061
**ycf46**	0.103	0.144	0.001	0.093	0.123	0.093	0.123
**ycf4**	0.084	0.001	0.001	0.085	0.080	0.106	0.099
**ycf52**	0.044	0.072	NA	0.045	0.044	0.045	0.044
**ycf53**	0.229	0.001	0.001	0.251	0.241	0.243	0.234
**ycf54**	0.174	0.001	0.001	0.177	0.125	0.272	0.172
**ycf55**	0.130	0.150	NA	0.148	0.137	0.148	0.137
**ycf56**	0.266	0.110	50	0.251	0.277	0.278	0.309
**ycf57**	0.033	0.001	0.001	0.036	0.030	0.039	0.031
**ycf60**	0.022	0.065	0.214	0.048	0.020	0.024	0.001
**ycf63**	0.045	0.117	0.079	0.037	0.047	0.044	0.059
**ycf65**	0.019	0.153	NA	0.001	0.038	0.001	0.038
**ycf80**	0.118	0.106	0.138	0.121	0.120	0.118	0.118
**ycf92**	0.290	0.372	0.475	0.318	0.268	0.317	0.271

Table S5. Result of dN/dS analysis of mtDNA genes.

	All	Korea /Oregon	Chile /UK	Chile /Korea	Chile /Oregon	UK /Korea	UK /Oregon
**Maturase**	NA	NA	NA	0.913	0.121	NA	0.116
**atp6**	0.008	0.275	0.017	50.000	0.006	0.011	0.001
**atp8**	0.168	0.283	0.182	1.293	0.171	0.167	0.155
**atp9**	0.001	0.001	0.001	0.001	0.001	0.001	0.001
**cob**	0.027	0.052	0.026	0.001	0.030	0.025	0.029
**cox1**	0.005	0.064	0.006	0.001	0.004	0.006	0.004
**cox2**	0.011	0.113	0.011	0.059	0.018	0.005	0.011
**cox3**	0.040	0.001	0.043	0.205	0.042	0.039	0.037
**orf729**	0.107	0.463	0.106	0.536	0.108	0.107	0.106
**nad1**	0.015	0.075	0.014	0.001	0.017	0.013	0.016
**nad2**	0.059	0.212	0.063	0.039	0.056	0.065	0.058
**nad3**	0.023	0.001	0.023	0.001	0.025	0.021	0.023
**nad4L**	0.014	0.101	0.009	0.001	0.016	0.010	0.018
**nad4**	0.037	0.353	0.039	0.071	0.038	0.036	0.035
**nad5**	0.064	0.072	0.065	0.082	0.061	0.067	0.064
**nad6**	0.075	0.339	0.083	1.140	0.096	0.058	0.068
**rpl16**	0.064	0.435	0.059	50.000	0.067	0.059	0.067
**rpl20**	0.312	0.214	0.290	50.000	0.279	0.312	0.305
**rps11**	0.120	0.001	0.119	50.000	0.131	0.119	0.131
**rps12**	0.001	0.001	0.001	0.001	0.001	0.001	0.001
**rps3**	0.083	0.051	0.081	0.001	0.085	0.078	0.082
**sdh2**	0.051	0.123	0.059	0.866	0.055	0.049	0.045
**sdh3**	0.262	50.000	0.239	0.434	0.238	0.274	0.272
**sdhD**	0.218	NA	0.219	NA	0.219	0.219	0.219
**secY**	0.153	0.207	0.149	0.162	0.152	0.157	0.161
**ymf39**	0.117	0.754	0.114	0.351	0.112	0.126	0.125

Table S6. Species and GeneBank Accession information used in concatenated ptDNA gene phylogeny.

Class	Subclass	Species	GeneBank Accession	Reference
**Bangiophyceae**		*Bangia fuscopurpurea*	KP714733	(Cao et al., 2018)
		Pyropia endiviifolia	KT716756	(Xu et al., 2018)
		Pyropia haitanensis	NC_021189	(Wang et al., 2013)
		Pyropia pulchra	NC_029861	(Lee et al., 2016b)
		Wildemania schizophylla	NC_029576	(Jeffery, 2016)
		Pyropia yezoensis	MK695880	(Xu et al., 2019)
		Pyropia yezoensis	KC517072	(Wang et al., 2013)
		Pyropia yezoensis	NC_007932	(Wang et al., 2013)
		Porphyra purpurea	NC_000925	(Reith and Munholland, 1995)
		Porphyra umbilicalis	NC_035573	(Brawley et al., 2017)
		Pyropia perforata	NC_024050	(Hughey et al., 2014)
**Florideophyceae**	Hildenbrandiophycidae	*Apophlaea sinclairii*	NC_031172	(Lee et al., 2016a)
		Hildenbrandia rubra	NC_031146	(Lee et al., 2016a)
		Hildenbrandia rivularis	NC_031177	(Lee et al., 2016a)
	Nemaliophycidae	*Acrochaetium secundatum*	NC_042170	(Evans et al., 2019)
		Balbiania investiens	NC_042171	(Evans et al., 2019)
		Dermonema virens	NC_031655	(F. Costa et al., 2016)
		Dichotomaria marginata	NC_031656	(F. Costa et al., 2016)
		Galaxaura rugosa	NC_031657	(F. Costa et al., 2016)
		Helminthocladia australis	NC_031658	(F. Costa et al., 2016)
		Helminthora furcellata	NC_031654	(F. Costa et al., 2016)
		Izziella formosana	NC_031660	(F. Costa et al., 2016)
		Kumanoa americana	NC_031178	(Cho et al., 2018)
		Liagora brachyclada	NC_031667	(F. Costa et al., 2016)
		Liagora harveyana	NC_031661	(F. Costa et al., 2016)
		Liagoropsis maxima	NC_031662	(F. Costa et al., 2016)
		Nemalion sp.	LT622871	(F. Costa et al., 2016)
		Neoizziella asiatica	NC_031663	(F. Costa et al., 2016)
		Palmaria decipiens	NC_046495	(Bustamante et al., 2020)
		Palmaria palmata	NC_031147	(Cho et al., 2018)

	Scinaia undulata	NC_031664	(F. Costa et al., 2016)
	Sheathia arcuata	NC_035231	(Nan et al., 2017)
	Thorea hispida	NC_031171	(Cho et al., 2018)
	Titanophycus setchellii	NC_031665	(F. Costa et al., 2016)
	Trichogloeopsis pedicellata	NC_031668	(F. Costa et al., 2016)
	Yamadaella caenomyce	NC_031666	(F. Costa et al., 2016)
Coreallinophycidae	*Calliarthron tuberculosum*	NC_021075	(Janouškovec et al., 2013)
	Corallina chilensis	NC_042901	(Alejo et al., 2019)
	Corallina ferreyrae	NC_041636	(Bustamante et al., 2019)
	Corallina officinalis	MT211886	(Yesson et al., 2020)
	Lithothamnion sp.	MH281627	(Lee et al., 2018)
	Neogoniolithon spectabile	NC_039978	(Lee et al., 2018)
	Porolithon onkodes	NC_038144	(Gabrielson et al., 2018)
	Renouxia sp.	MH281629	(Lee et al., 2018)
	Sporolithon durum	NC_029857	(Lee et al., 2016b)
	Synarthrophyton chejuense	NC_039977	(Lee et al., 2018)
Rhodymeniophycidae	*Acrosorium ciliolatum*	NC_035260	(Díaz-Tapia et al., 2017)
	Agarophyton chilense	NC_029860	(Lee et al., 2016b)
	Agarophyton vermiculophyllum	NC_039092	(Iha et al., 2018)
	Asparagopsis taxiformis	NC_031148	(Lee et al., 2016a)
	Bostrychia moritziana	NC_035266	(Díaz-Tapia et al., 2017)
	Bostrychia simpliciuscula	NC_035268	(Díaz-Tapia et al., 2017)
	Bostrychia tenella	NC_035264	(Díaz-Tapia et al., 2017)
	Bryothamnion seaforthii	NC_035276	(Díaz-Tapia et al., 2017)
	Caloglossa beccarii	NC_035269	(Díaz-Tapia et al., 2017)
	Caloglossa intermedia	NC_035265	(Díaz-Tapia et al., 2017)
	Caloglossa monosticha	NC_035263	(Díaz-Tapia et al., 2017)
	Caulacanthus okamurae	NC_047434	(Aguilar et al., 2020)
	Ceramium japonicum	NC_031174	(Lee et al., 2016a)
	Ceramium sungminbooi	NC_031211	(Hughey and Boo, 2016)
	Chondria sp.	MF101451	(Díaz-Tapia et al., 2017)
	Chondrus crispus	NC_020795	(Collén et al., 2013)
	Cliftonaea pectinata	NC_035294	(Díaz-Tapia et al., 2017)

	Coeloseira compressa	NC_030338	(Kilpatrick and Hughey, 2016)
	Crassa caudata	NC_039139	(Iha et al., 2018)
	Crassa firma	NC_033877	(Ng et al., 2017)
	Dasya binghamiae	NC_031161	(Tamayo and Hughey, 2016)
	Dasya naccarioides	NC_035280	(Díaz-Tapia et al., 2017)
	Dasyclonium flaccidum	NC_035287	(Díaz-Tapia et al., 2017)
	Dictyomenia sonderi	NC_035297	(Díaz-Tapia et al., 2017)
	Digenea simplex	NC_035298	(Díaz-Tapia et al., 2017)
	Dipterocladia arabiensis	NC_035257	(Díaz-Tapia et al., 2017)
	Dipterosiphonia australica	NC_035288	(Díaz-Tapia et al., 2017)
	Gelidium coulteri	NC_041173	(Boo and Hughey, 2019)
	Gelidium elegans	NC_029858	(Lee et al., 2016b)
	Gelidium gabrielsonii	NC_040156	(Boo and Hughey, 2019)
	Gelidium galapagense	NC_041174	(Boo and Hughey, 2019)
	Gelidium kathyanniae	NC_040158	(Boo and Hughey, 2019)
	Gelidium sinicola	NC_041175	(Boo and Hughey, 2019)
	Gelidium vagum	NC_029859	(Lee et al., 2016b)
	Gracilaria spinulosa	NC_046042	(Liu et al., 2019c)
	Gracilaria edulis	NC_046041	(Liu et al., 2019b)
	Gracilaria ferox	NC_039140	(Iha et al., 2018)
	Gracilaria gracilis	NC_039141	(Iha et al., 2018)
	Gracilaria salicornia	NC_023785	(Campbell et al., 2014)
	Gracilaria tenuistipitata var. *liui*	AY673996	(Hagopian et al., 2004)
	Gracilaria textorii	NC_046043	(Chen et al., 2019)
	Gracilariopsis chorda	KX284722	(Lee et al., 2016a)
	Gracilariopsis heteroclada	NC_038100	-
	Gracilariopsis lemaneiformis	KP330491	(Du et al., 2016)
	Gracilariopsis lemaneiformis	NC_029644	(Zhang et al., 2016)
	Gracilariopsis longissima	NC_039143	(Iha et al., 2018)
	Gracilariopsis mclachlanii	NC_039144	(Iha et al., 2018)
	Grateloupia filicina	NC_037841	(Zhang et al., 2018)
	Grateloupia taiwanensis	NC_021618	(DePriest et al., 2013)
	Grateloupia turuturu	MN853877	(Han and Li, 2020)
	Gredgaria maugeana	NC_035290	(Díaz-Tapia et al., 2017)

	Herposiphonia versicolor	NC_035279	(Díaz-Tapia et al., 2017)
	Hydropuntia rangiferina	NC_039142	(Iha et al., 2018)
	Kappaphycus alvarezii	KU892652	(Liu et al., 2019a)
	Kuetzingia canaliculata	NC_035293	(Díaz-Tapia et al., 2017)
	Laurencieae sp.	MF101412	(Díaz-Tapia et al., 2017)
	Laurenciella marilzae	NC_035259	(Díaz-Tapia et al., 2017)
	Leptosiphonia brodiei	NC_035272	(Díaz-Tapia et al., 2017)
	Lophocladia kuetzingii	NC_035292	(Díaz-Tapia et al., 2017)
	Mastocarpus papillatus	NC_031167	(Sissini et al., 2016)
	Melanothamnus harveyi	NC_035281	(Díaz-Tapia et al., 2017)
	Melanthalia intermedia	NC_039145	(Iha et al., 2018)
	Membranoptera platyphylla	NC_032041	(Hughey et al., 2017)
	Membranoptera tenuis	NC_032399	(Hughey et al., 2017)
	Membranoptera weeksiae	NC_032396	(Hughey et al., 2017)
	Schizymenia dubyi	NC_031169	(Lee et al., 2016a)
	Ophidocladus simpliciusculus	NC_035284	(Díaz-Tapia et al., 2017)
	Osmundaria fimbriata	NC_035262	(Díaz-Tapia et al., 2017)
	Palisada sp.	MF101453	(Díaz-Tapia et al., 2017)
	Periphykon beckeri	NC_035261	(Díaz-Tapia et al., 2017)
	Platysiphonia delicata	NC_035258	(Díaz-Tapia et al., 2017)
	Pleurostichidium falkenbergii	NC_042794	(Pasella et al., 2019)
	Plocamium cartilagineum	NC_031179	(Lee et al., 2016a)
	Polysiphonia sp.	MF101414	(Díaz-Tapia et al., 2017)
	Polysiphonia sp.	MF101456	(Díaz-Tapia et al., 2017)
	Polysiphonia elongata	NC_035274	(Díaz-Tapia et al., 2017)
	Polysiphonia infestans	NC_035277	(Díaz-Tapia et al., 2017)
	Polysiphonia schneideri	NC_035296	(Díaz-Tapia et al., 2017)
	Polysiphonia scopulorum	NC_035282	(Díaz-Tapia et al., 2017)
	Polysiphonia sertularioides	NC_035270	(Díaz-Tapia et al., 2017)
	Polysiphonia stricta	NC_035275	(Díaz-Tapia et al., 2017)
	Pterocladia lucida	MT117916	(Preuss et al., 2020)
	Rhodomela confervoides	NC_035271	(Díaz-Tapia et al., 2017)

	Rhodymenia pseudopalmata	NC_031144	(Lee et al., 2016a)
	Riquetophycus sp.	KX284710	(Lee et al., 2016a)
	Schimmelmannia schousboei	NC_031168	(Lee et al., 2016a)
	Sebdenia flabellata	NC_031170	(Lee et al., 2016a)
	Sonderella linearis	NC_035289	(Díaz-Tapia et al., 2017)
	Spyridia filamentosa	NC_035285	(Díaz-Tapia et al., 2017)
	Symphyocladia dendroidea	NC_035267	(Díaz-Tapia et al., 2017)
	Taenioma perpusillum	NC_035295	(Díaz-Tapia et al., 2017)
	Thaumatella adunca	NC_035291	(Díaz-Tapia et al., 2017)
	Thuretia quercifolia	NC_035286	(Díaz-Tapia et al., 2017)
	Tolypiocladia glomerulata	NC_035299	(Díaz-Tapia et al., 2017)
	Vertebrata australis	NC_035283	(Díaz-Tapia et al., 2017)
	Vertebrata isogona	NC_035278	(Díaz-Tapia et al., 2017)
	Vertebrata lanosa	NC_026523	(Salomaki et al., 2015)
	Vertebrata thuyoides	NC_035273	(Díaz-Tapia et al., 2017)

Table S7. Species and GeneBank Accession information used in concatenated mtDNA gene phylogeny.

Class	Subclass	Species	GeneBank Accession	Reference
Bangiophyceae		*Bangia fuscopurpurea*	NC_026905	-
		Porphyra purpurea	NC_002007	(Burger et al., 1999)
		Pyropia endiviifolia	KU356193	(Xu et al., 2018)
		Pyropia fucicola	NC_024288	(Hughey et al., 2014)
		Pyropia haitanensis	NC_017751	(Mao et al., 2012)
		Pyropia kanakaensis	NC_024289	(Hughey et al., 2014)
		Pyropia nitida	NC_027616	(Harden et al., 2016)
		Pyropia perforata	KF515974	(Hughey et al., 2014)
		Pyropia perforata	KF515975	(Hughey et al., 2014)
		Pyropia perforata	KJ708766	(Hughey et al., 2014)
		Pyropia pulchra	MT588076	(Park and Lee, 2020)
		Pyropia tenera	NC_021475	(Hwang et al., 2013)
		Pyropia yezoensis	KF561997	(Hwang et al., 2014)
		Pyropia yezoensis	NC_017837	(Kong et al., 2014)
		Wildemania schizophylla	NC_024579	(Silva and Hughey, 2016)
Florideophyceae	Hildenbrandiophycidae	*Hildenbrandia rubra*	NC_026055	(Yang et al., 2015)
	Nemaliophycidae	*Balbiania investiens*	NC_042172	(Evans et al., 2019)
		Kumanoa ambigua	NC_039405	(Paiano et al., 2018)
		Kumanoa mahlacensis	NC_039406	(Paiano et al., 2018)
		Lympha mucosa	NC_036231	(Wolf et al., 2017)
		Palmaria decipiens	NC_046496	(Bustamante et al., 2020)
		Palmaria palmata	AP019296	(Kumagai et al., 2019)
		Palmaria palmata	NC_026056	(Yang et al., 2015)
		Paralemanea sp.	MG787097	(Paiano et al., 2018)
		Sheathia arcuata	NC_035349	(Nan et al., 2017)
		Sirodotia delicatula	NC_039407	(Paiano et al., 2018)
		Thorea hispida	NC_035158	(Nan et al., 2017)
	Coreallinophycidae	*Calliarthron tuberculosum*	NC_027061	(Bi et al., 2016)
		Corallina chilensis	NC_042900	(Alejo et al., 2019)

		Corallina ferreyrae	NC_041635	(Bustamante et al., 2019)
		Corallina officinalis	MT211883	(Yesson et al., 2020)
		Corallina officinalis	NC_033904	(Williamson et al., 2016)
		Lithothamnion sp.	MH281621	(Lee et al., 2018)
		Neogoniolithon spectabile	NC_039980	(Lee et al., 2018)
		Porolithon onkodes	NC_038145	(Gabrielson et al., 2018)
		Renouxia sp.	MH281622	(Lee et al., 2018)
		Rhodogorgon sp.	MH281625	(Lee et al., 2018)
		Sporolithon durum	NC_023454	(Kim et al., 2015)
		Synarthrophyton chejuense	NC_039979	(Lee et al., 2018)
	Rhodymeniophycidae	*Agarophyton chilense*	MF401962	(Liu et al., 2017)
		Agarophyton tenuistipitatum	NC_037891	(Liu et al., 2018)
		Agarophyton vermiculophyllum	KJ526626	-
		Agarophyton vermiculophyllum	MH396022	(Iha et al., 2018)
		Asparagopsis taxiformis	NC_026843	(Yang et al., 2015)
		Betaphycus gelatinus	NC_036431	(Li et al., 2018a)
		Caulacanthus okamurae	NC_047435	(Aguilar et al., 2020)
		Ceramium japonicum	KJ398159	(Yang et al., 2015)
		Chondrus crispus	NC_001677	(Burger et al., 1999)
		Choreocolax polysiphoniae	NC_032002	(Salomaki and Lane, 2017)
		Crassa caudata	NC_039146	(Iha et al., 2018)
		Dasya binghamiae	NC_031160	(Tamayo and Hughey, 2016)
		Eucheuma denticulatum	NC_036432	(Li et al., 2018a)
		Gelidium arborescens	NC_031836	(Boo et al., 2016)
		Gelidium coulteri	NC_040985	-
		Gelidium crinale f. *luxurians*	KX427229	(Boo et al., 2016)
		Gelidium elegans	NC_026053	(Yang et al., 2015)
		Gelidium gabrielsonii	NC_040155	(Boo et al., 2016)
		Gelidium galapagense	KX427230	(Boo et al., 2016)
		Gelidium isabelae	NC_031838	(Boo et al., 2016)
		Gelidium kathyanniae	NC_040157	(Boo et al., 2016)

	Gelidium sclerophyllum	NC_031839	(Boo et al., 2016)
	Gelidium sinicola	KX427233	(Boo et al., 2016)
	Gelidium vagum	NC_023077	(Yang et al., 2014a)
	Gloiopeltis furcata	NC_044414	(Watanabe et al., 2019)
	Gracilaria changii	NC_034681	(Song et al., 2017)
	Gracilaria chouae	NC_038211	(Tang et al., 2019)
	Gracilaria edulis	MG592725	(Sedanza et al., 2020)
	Gracilaria ferox	NC_039147	(Iha et al., 2018)
	Gracilaria gracilis	NC_039148	(Iha et al., 2018)
	Gracilaria salicornia	NC_023784	(Campbell et al., 2014)
	Gracilaria spinulosa	NC_037890	(Wang et al., 2019)
	Gracilaria tenuistipitata var. *liui*	MG592728	(Tang et al., 2018)
	Gracilaria textorii	MG592729	(Yuan, 2018)
	Gracilariophila oryzoides	NC_014771	(Hancock et al., 2010)
	Gracilariopsis andersonii	NC_014772	(Hancock et al., 2010)
	Gracilariopsis chorda	NC_023251	(Yang et al., 2014b)
	Gracilariopsis heteroclada	NC_038101	-
	Gracilariopsis longissima	NC_039150	(Iha et al., 2018)
	Gracilariopsis mclachlanii	NC_039151	(Iha et al., 2018)
	Grateloupia angusta	NC_023094	(Kim et al., 2014b)
	Grateloupia filicina	NC_037842	(Li et al., 2018b)
	Grateloupia taiwanensis	KM999231	(DePriest et al., 2014)
	Hydropuntia rangiferina	NC_039149	(Iha et al., 2018)
	Kappaphycus alvarezii	NC_031814	(Li et al., 2018a)
	Kappaphycus striatus	NC_024265	(Tablizo and Lluisma, 2014)
	Mastocarpus papillatus	NC_031166	(Sissini et al., 2016)
	Melanthalia intermedia	NC_039152	(Iha et al., 2018)
	Plocamiocolax pulvinata	NC_014773	(Hancock et al., 2010)
	Pterocladia lucida	MT117917	(Preuss et al., 2020)
	Pterocladia mexicana	NC_031842	(Boo et al., 2016)
	Pterocladia robusta	NC_031844	(Boo et al., 2016)

	Pterocladiella media	NC_031841	(Boo et al., 2016)
	Pterocladiella musciformis	NC_031843	(Boo et al., 2016)
	Pterocladiophila hemisphaerica	MT117919	(Preuss et al., 2020)
	Rhodymenia pseudopalmata	NC_023252	(Kim et al., 2014a)
	Vertebrata lanosa	NC_032003	(Salomaki and Lane, 2017)

Supplementary References

Aguilar, A., Ahumada, T.J., Amezcua Moreno, N., Bohn, J., Bustamante, D.E., Calderon, M.S., Cardoso, E., Carranza, R., Castillo, M., Cazares, E., Cazares, E., Companion, J.K., Cruz, J., Cuevas, N., De La Torre, L., Dietz, D.P., Fernando, K.M., Garcia, B., Gomez, P., Gonzales-Miramontes, B., Hernandez, Y., Huaracha, K., Hughey, J.R., Lazaro, G., Zhai Lorenzo, F., Medrano, D., Mendoza, A., Mendoza, D., Mohssin, A., Orozco Medina, J., Pacheco, A., Palacios Ruvalcaba, G., Patel, J., Patel, J., Patino, S., Perez-Alfaro, K., Ponce, A.N., Poso, J.G., Ramirez, G., Ramirez, H.A., Resendiz, N., Reyno, R., Rodriguez, D., Russell, I.A., Saenz-Verdugo, P., Carmona, A.S., Sanchez, F., Sheffer, S.X., Solorio, C., Soto Trujillo, A., Vasaya, G.S., and Velasquez Lopez, V. (2020). The complete mitochondrial and plastid genomes of the invasive marine red alga *Caulacanthus okamurae* (Caulacanthaceae, Rhodophyta) from Moss Landing, California, USA. *Mitochondrial DNA Part B* 5**,** 2067-2069.

Alejo, I.A., Aleman, T.E., Almanza, K., Alonso, W., Altamirano Manriquez, M.G., Armbrister, T., Astudillo, Y., Batistiana, L., Blas Guido, J.S., Bustamante, D.E., Calderon, M.S., Camacho Gonzalez, J.D., Cardoso, S., Castro, J., Chombo Garcia, M.N., Colin, L.P., Cortina, K.G., Delgado, A., Espinoza Castro, D., Estrada, I., Felix, E., Felix, S., Flores, M., Frausto, S., Garcia, E.D., Garcia, M., Gasca, G., Gomez, D., Gonzalez Balcazar, C., Miramontes, B.G., Gonzalez, V., Guzman, C.D., Guzman, E.C., Hanneman, T.B., Hernandez, J., Hughey, J.R., Hutchins, V.N., Kallison, E.R., Lepe, S., Lopez, S.M., Zhai Lorenzo, F., Macias Reyes, E., Madrigal, F., Madrigal-Gonzalez, N.G., Mandujano, R., Manzo, R., Martinez, P.L., Martinez, S., Medina, B.A., Mendez, M., Mendoza Contreras, J.J., Meza, A.I., Miller, K., Morales, A., Munoz, E., Myers, J.M., Patel, P.-A., Montes, D.P., Ponce, A.N., Ramirez, A.E., Rico, E., Rodriguez, A., Rodriguez, J., Ruiz, A., Saldana, A., Sanchez, R.A., Santana, J.D., Solano, F.D., Soto Trujillo, A., Soto, S.J., Steinhardt, A., Talavera, M., Tapia, M.M., Tapia, O., Taveras Dina, M.O., Torresillas, B.J., Vazquez-Ramos, C., and Wong, F.L. (2019). The complete mitochondrial and plastid genomes of *Corallina chilensis* (Corallinaceae, Rhodophyta) from Tomales Bay, California, USA. *Mitochondrial DNA Part B* 4**,** 1879-1880.

Bi, G., Liu, G., Zhao, E., and Du, Q. (2016). Complete mitochondrial genome of a red calcified alga *Calliarthron tuberculosum* (Corallinales). *Mitochondrial DNA Part A* 27**,** 2554-2556.

Boo, G.H., and Hughey, J.R. (2019). Phylogenomics and multigene phylogenies decipher two new cryptic marine algae from California, *Gelidium gabrielsonii* and *G. kathyanniae* (Gelidiales, Rhodophyta). *J. Phycol.* 55**,** 160-172.

Boo, G.H., Hughey, J.R., Miller, K.A., and Boo, S.M. (2016). Mitogenomes from type specimens, a genotyping tool for morphologically simple species: ten genomes of agar-producing red algae. *Sci. Rep.* 6**,** 35337.

Brawley, S.H., Blouin, N.A., Ficko-Blean, E., Wheeler, G.L., Lohr, M., Goodson, H.V., Jenkins, J.W., Blaby-Haas, C.E., Helliwell, K.E., Chan, C.X., Marriage, T.N., Bhattacharya, D., Klein, A.S., Badis, Y., Brodie, J., Cao, Y., Collén, J., Dittami, S.M., Gachon, C.M.M., Green, B.R., Karpowicz, S.J., Kim, J.W., Kudahl, U.J., Lin, S., Michel, G., Mittag, M., Olson, B.J.S.C., Pangilinan, J.L., Peng, Y., Qiu, H., Shu, S., Singer, J.T., Smith, A.G., Sprecher, B.N., Wagner, V., Wang, W., Wang, Z.-Y., Yan, J., Yarish, C., Zäuner-Riek, S., Zhuang, Y., Zou, Y., Lindquist, E.A., Grimwood, J., Barry, K.W., Rokhsar, D.S., Schmutz, J., Stiller, J.W., Grossman, A.R., and Prochnik, S.E. (2017). Insights into the red algae and eukaryotic evolution from the genome of *Porphyra umbilicalis* (Bangiophyceae, Rhodophyta). *Proc. Natl. Acad. Sci. U.S.A.* 114**,** E6361.

Bringloe, T.T., and Saunders, G.W. (2019). Trans-Arctic speciation of Florideophyceae (Rhodophyta) since the opening of the Bering Strait, with consideration of the “species pump” hypothesis. *J. Biogeogr.* 46, 694-705.

Burger, G., Saint-Louis, D., Gray, M.W., and Lang, B.F. (1999). Complete sequence of the mitochondrial DNA of the red alga *Porphyra purpurea*: cyanobacterial introns and shared ancestry of red and green algae. *The Plant Cell* 11**,** 1675.

Bustamante, D.E., Calderon, M.S., and Hughey, J.R. (2019). Conspecificity of the Peruvian *Corallina ferreyrae* with *C. caespitosa* (Corallinaceae, Rhodophyta) inferred from genomic analysis of the type specimen. *Mitochondrial DNA Part B* 4**,** 1285-1286.

Bustamante, D.E., Hughey, J.R., Calderon, M.S., Mansilla, A., Rodriguez, J.P., and Mendez, F. (2020). Analysis of the complete organellar genomes of *Palmaria decipiens* (Palmariaceae, Rhodophyta) from Antarctica confirms its taxonomic placement in the genus *Palmaria*. *Mitochondrial DNA Part B* 5**,** 1327-1328.

Campbell, M.A., Presting, G., Bennett, M.S., and Sherwood, A.R. (2014). Highly conserved organellar genomes in the Gracilariales as inferred using new data from the Hawaiian invasive alga *Gracilaria salicornia* (Rhodophyta). *Phycologia* 53, 109-116.

Cao, M., Bi, G., Mao, Y., Li, G., and Kong, F. (2018). The first plastid genome of a filamentous taxon ‘*Bangia*’ sp. OUCPT-01 in the Bangiales. *Sci. Rep.* 8**,** 10688.

Chen, W., Liu, T., Tang, X., Jia, X., and Wu, X. (2019). The complete plastid genome and phylogenetic analysis of *Gracilaria textorii*. *Mitochondrial DNA Part B* 4**,** 2608-2609.

Cho, C.H., Choi, J.W., Lam, D.W., Kim, K.M., and Yoon, H.S. (2018). Plastid genome analysis of three Nemaliophycidae red algal species suggests environmental adaptation for iron limited habitats. *PLOS ONE* 13**,** e0196995.

Collén, J., Porcel, B., Carré, W., Ball, S.G., Chaparro, C., Tonon, T., Barbeyron, T., Michel, G., Noel, B., Valentin, K., Elias, M., Artiguenave, F., Arun, A., Aury, J.-M., Barbosa-Neto, J.F., Bothwell, J.H., Bouget, F.-Y., Brillet, L., Cabello-Hurtado, F., Capella-Gutiérrez, S., Charrier, B., Cladière, L., Cock, J.M., Coelho, S.M., Colleoni, C., Czjzek, M., Da Silva, C., Delage, L., Denoeud, F., Deschamps, P., Dittami, S.M., Gabaldón, T., Gachon, C.M.M., Groisillier, A., Hervé, C., Jabbari, K., Katinka, M., Kloareg, B., Kowalczyk, N., Labadie, K., Leblanc, C., Lopez, P.J., Mclachlan, D.H., Meslet-Cladiere, L., Moustafa, A., Nehr, Z., Nyvall Collén, P., Panaud, O., Partensky, F., Poulain, J., Rensing, S.A., Rousvoal, S., Samson, G., Symeonidi, A., Weissenbach, J., Zambounis, A., Wincker, P., and Boyen, C. (2013). Genome structure and metabolic features in the red seaweed *Chondrus crispus* shed light on evolution of the Archaeplastida. *Proc. Natl. Acad. Sci. U.S.A.* 110**,** 5247.

Depriest, M.S., Bhattacharya, D., and López-Bautista, J.M. (2013). The plastid genome of the red macroalga *Grateloupia taiwanensis* (Halymeniaceae). *PLOS ONE* 8**,** e68246.

Depriest, M.S., Bhattacharya, D., and López-Bautista, J.M. (2014). The mitochondrial genome of *Grateloupia taiwanensis* (Halymeniaceae, Rhodophyta) and comparative mitochondrial genomics of red algae. *Biol. Bull.* 227**,** 191-200.

Díaz-Tapia, P., Maggs, C.A., West, J.A., and Verbruggen, H. (2017). Analysis of chloroplast genomes and a supermatrix inform reclassification of the Rhodomelaceae (Rhodophyta). *J. Phycol.* 53**,** 920-937.

Dixon, K.R., and Saunders, G.W. (2013). DNA barcoding and phylogenetics of *Ramicrusta* and *Incendia* gen. nov., two early diverging lineages of the Peyssonneliaceae (Rhodophyta). *Phycologia* 52, 82-108.

Du, Q., Bi, G., Mao, Y., and Sui, Z. (2016). The complete chloroplast genome of *Gracilariopsis lemaneiformis* (Rhodophyta) gives new insight into the evolution of family Gracilariaceae. *J. Phycol.* 52**,** 441-450.

Evans, J.R., St. Amour, N., Verbruggen, H., Salomaki, E.D., and Vis, M.L. (2019). Chloroplast and mitochondrial genomes of *Balbiania investiens* (Balbianiales, Nemaliophycidae). *Phycologia* 58, 310-318.

F. Costa, J., Lin, S.M., Macaya, E.C., Fernández-García, C., and Verbruggen, H. (2016). Chloroplast genomes as a tool to resolve red algal phylogenies: a case study in the Nemaliales. *BMC Evol. Biol.* 16**,** 205.

Freshwater, D.W., Fredericq, S., Butler, B.S., Hommersand, M.H., and Chase, M.W. (1994). A gene phylogeny of the red algae (Rhodophyta) based on plastid *rbc*L. *Proc. Natl. Acad. Sci. U.S.A.* 91, 7281.

Gabrielson, P.W., Hughey, J.R., and Diaz-Pulido, G. (2018). Genomics reveals abundant speciation in the coral reef building alga *Porolithon onkodes* (Corallinales, Rhodophyta). *J. Phycol.* 54**,** 429-434.

Hagopian, J.C., Reis, M., Kitajima, J.P., Bhattacharya, D., and De Oliveira, M.C. (2004). Comparative analysis of the complete plastid genome sequence of the red alga *Gracilaria tenuistipitata* var. *liui* provides insights into the evolution of rhodoplasts and their relationship to other plastids. *J. Mol. Evol.* 59**,** 464-477.

Han, H., and Li, Y. (2020). The complete chloroplast genome of *Grateloupia turuturu* Yamada. *Mitochondrial DNA Part B* 5**,** 1087-1089.

Hancock, L., Goff, L., and Lane, C. (2010). Red algae lose key mitochondrial genes in response to becoming parasitic. *Genome Biol. Evol.* 2**,** 897-910.

Harden, L.K., Morales, K.M., and Hughey, J.R. (2016). Identification of a new marine algal species *Pyropia nitida* sp. nov. (Bangiales: Rhodophyta) from Monterey, California. *Mitochondrial DNA Part A* 27**,** 3058-3062.

Hughey, J.R., and Boo, G.H. (2016). Genomic and phylogenetic analysis of *Ceramium cimbricum* (Ceramiales, Rhodophyta) from the Atlantic and Pacific Oceans supports the naming of a new invasive Pacific entity *Ceramium sungminbooi* sp. nov. *Bot. Mar.* 59**,** 211-222.

Hughey, J.R., Gabrielson, P.W., Rohmer, L., Tortolani, J., Silva, M., Miller, K.A., Young, J.D., Martell, C., and Ruediger, E. (2014). Minimally destructive sampling of type specimens of *Pyropia* (Bangiales, Rhodophyta) recovers complete plastid and mitochondrial genomes. *Sci. Rep.* 4**,** 5113.

Hughey, J.R., Hommersand, M.H., Gabrielson, P.W., Miller, K.A., and Fuller, T. (2017). Analysis of the complete plastomes of three species of *Membranoptera* (Ceramiales, Rhodophyta) from Pacific North America. *J. Phycol.* 53**,** 32-43.

Hwang, M.S., Kim, S.O., Ha, D.S., Lee, J.E., and Lee, S.R. (2013). Complete sequence and genetic features of the mitochondrial genome of *Pyropia tenera* (Rhodophyta). *Plant Biotechnol. Rep.* 7**,** 435-443.

Hwang, M.S., Kim, S.O., Ha, D.-S., Lee, J.E., and Lee, S.R. (2014). Complete mitochondrial genome sequence of *Pyropia yezoensis* (Bangiales, Rhodophyta) from Korea. *Plant Biotechnol. Rep.* 8**,** 221-227.

Iha, C., Grassa, C.J., Lyra, G.D.M., Davis, C.C., Verbruggen, H., and Oliveira, M.C. (2018). Organellar genomics: a useful tool to study evolutionary relationships and molecular evolution in Gracilariaceae (Rhodophyta). *J. Phycol.* 54**,** 775-787.

Janouškovec, J., Liu, S.L., Martone, P.T., Carré, W., Leblanc, C., Collén, J., and Keeling, P.J. (2013). Evolution of red algal plastid genomes: ancient architectures, introns, horizontal gene transfer, and taxonomic utility of plastid markers. *PLOS ONE* 8**,** e59001.

Jeffery, R.H. (2016). Genomic and phylogenetic analysis of the complete plastid genome of the California endemic seaweed *Wildemania schizophylla* (Bangiaceae). *Madroño* 63**,** 34-38.

Kilpatrick, Z.M., and Hughey, J.R. (2016). Mitochondrial and plastid genome analysis of the marine red alga *Coeloseira compressa* (Champiaceae, Rhodophyta). *Mitochondrial DNA Part B* 1, 456-458.

Kim, K.M., Yang, E.C., Kim, J.H., Nelson, W.A., and Yoon, H.S. (2015). Complete mitochondrial genome of a rhodolith, *Sporolithon durum* (Sporolithales, Rhodophyta). *Mitochondrial DNA* 26, 155-156.

Kim, K.M., Yang, E.C., Yi, G., and Yoon, H.S. (2014a). Complete mitochondrial genome of sublittoral macroalga *Rhodymenia pseudopalmata* (Rhodymeniales, Rhodophyta). *Mitochondrial DNA* 25, 273-274.

Kim, S.Y., Yang, E.C., Boo, S.M., and Yoon, H.S. (2014b). Complete mitochondrial genome of the marine red alga *Grateloupia angusta* (Halymeniales). *Mitochondrial DNA* 25, 269-270.

Kong, F., Sun, P., Cao, M., Wang, L., and Mao, Y. (2014). Complete mitochondrial genome of *Pyropia yezoensis*: reasserting the revision of genus *Porphyra*. *Mitochondrial DNA* 25, 335-336.

Kumagai, Y., Ryota, T., Miyabe, Y., Takeda, T., Adachi, K., Yasui, H., and Kishimura, H. (2019). Complete sequence of mitochondrial DNA of red alga dulse *Palmaria palmata* (Linnaeus) Weber & Mohr in Japan. *Mitochondrial DNA Part B* 4, 3177-3178.

Lee, J., Cho, C.H., Park, S.I., Choi, J.W., Song, H.S., West, J.A., Bhattacharya, D., and Yoon, H.S. (2016a). Parallel evolution of highly conserved plastid genome architecture in red seaweeds and seed plants. *BMC Biol.* 14, 75.

Lee, J., Kim, K.M., Yang, E.C., Miller, K.A., Boo, S.M., Bhattacharya, D., and Yoon, H.S. (2016b). Reconstructing the complex evolutionary history of mobile plasmids in red algal genomes. *Sci. Rep.* 6, 23744.

Lee, J.M., Song, H.J., Park, S.I., Lee, Y.M., Jeong, S.Y., Cho, T.O., Kim, J.H., Choi, H.G., Choi, C.G., Nelson, W.A., Fredericq, S., Bhattacharya, D., and Yoon, H.S. (2018). Mitochondrial and plastid genomes from coralline red algae provide insights into the incongruent evolutionary histories of organelles. *Genome Biol. Evol.* 10, 2961-2972.

Li, Y., Liu, N., Wang, X., Tang, X., Zhang, L., Meinita, M.D.N., Wang, G., Yin, H., Jin, Y., Wang, H., Liu, C., Chi, S., Liu, T., and Zhang, J. (2018a). Comparative genomics and systematics of *Betaphycus*, *Eucheuma*, and *Kappaphycus* (Solieriaceae: Rhodophyta) based on mitochondrial genome. *J. Appl. Phycol.* 30, 3435-3443.

Li, Y., Meinita, M.D.N., Liu, T., Chi, S., and Yin, H. (2018b). Complete sequences of the mitochondrial DNA of the *Grateloupia filicina* (Rhodophyta). *Mitochondrial DNA Part B* 3, 76-77.

Liu, N., Li, Y., Liu, C., Liu, T., and Chen, W. (2018). Complete sequence of mitochondrial DNA of *Gracilaria tenuistipitata* (Rhodophyta). *Mitochondrial DNA Part B* 3, 814-815.

Liu, N., Wang, G., Li, Y., Zhang, L., Meinita, M.D.N., Chen, W., Liu, T., and Chi, S. (2017). The complete mitochondrial genome of the economic red alga, *Gracilaria chilensis*. *Mitochondrial DNA Part B* 2, 716-717.

Liu, N., Zhang, L., Tang, X., Wang, X., Meinita, M.D.N., Wang, G., Chen, W., and Liu, T. (2019a). Complete plastid genome of *Kappaphycus alvarezii*: insights of large-scale rearrangements among Florideophyceae plastid genomes. *J. Appl. Phycol.* 31, 3997-4005.

Liu, T., Tang, X., Jia, X., Wu, X., Huang, M., Zeng, J., and Chen, W. (2019b). The complete plastid genome and phylogenetic analysis of *Gracilaria edulis*. *Mitochondrial DNA Part B* 4, 2598-2599.

Liu, T., Tang, X., Jia, X., Wu, X., Huang, M., Zeng, J., and Chen, W. (2019c). The complete plastid genome and phylogenetic analysis of *Gracilaria spinulosa*. *Mitochondrial DNA Part B* 4, 2606-2607.

Mao, Y., Zhang, B., Kong, F., and Wang, L. (2012). The complete mitochondrial genome of *Pyropia haitanensis* Chang et Zheng. *Mitochondrial DNA* 23**,** 344-346.

Nan, F., Feng, J., Lv, J., Liu, Q., Fang, K., Gong, C., and Xie, S. (2017). Origin and evolutionary history of freshwater Rhodophyta: further insights based on phylogenomic evidence. *Sci. Rep.* 7**,** 2934.

Ng, P.K., Lin, S.M., Lim, P.E., Liu, L.C., Chen, C.M., and Pai, T.W. (2017). Complete chloroplast genome of *Gracilaria firma* (Gracilariaceae, Rhodophyta), with discussion on the use of chloroplast phylogenomics in the subclass Rhodymeniophycidae. *BMC Genomics* 18**,** 40.

Paiano, M.O., Del Cortona, A., Costa, J.F., Liu, S.L., Verbruggen, H., De Clerck, O., and Necchi, O. (2018). Complete mitochondrial genomes of six species of the freshwater red algal order Batrachospermales (Rhodophyta). *Mitochondrial DNA Part B* 3**,** 607-610.

Park, S.I., and Lee, J. (2020). The complete mitochondrial genome of *Pyropia pulchra* (Bangiophyceae, Rhodophyta). *Mitochondrial DNA Part B* 5**,** 3157-3158.

Pasella, M.M., Verbruggen, H., Nelson, W.A., and Díaz-Tapia, P. (2019). The phylogenetic position of the morphologically unusual *Pleurostichidium falkenbergii* (Rhodomelaceae, Rhodophyta) based on plastid phylogenomics. *Phycologia* 58**,** 319-325.

Preuss, M., Verbruggen, H., and Zuccarello, G.C. (2020). The organelle genomes in the photosynthetic red algal parasite *Pterocladiophila hemisphaerica* (Florideophyceae, Rhodophyta) have elevated substitution rates and extreme gene loss in the plastid genome. *J. Phycol.* 56**,** 1006-1018.

Reith, M., and Munholland, J. (1995). Complete nucleotide sequence of the *Porphyra purpurea* chloroplast genome. *Plant Mol. Biol. Rep.* 13**,** 333-335.

Salomaki, E.D., and Lane, C.E. (2017). Red algal mitochondrial genomes are more complete than previously reported. *Genome Biol. Evol.* 9**,** 48-63.

Salomaki, E.D., Nickles, K.R., and Lane, C.E. (2015). The ghost plastid of *Choreocolax polysiphoniae*. *J. Phycol.* 51**,** 217-221.

Sedanza, M.G.C., Meinita, M.D.N., Tang, X., Chen, W., Yin, H., Liu, C., Jin, Y., Chi, S., Li, Y., and Liu, T. (2020). Complete sequence of mitochondrial DNA of *Gracilaria edulis* (Rhodophyta). *Mitochondrial DNA Part B* 5**,** 1128-1129.

Silva, M.Y., and Hughey, J.R. (2016). Complete mitochondrial genome of the holotype specimen of *Wildemania schizophylla* (Bangiales: Rhodophyta). *Mitochondrial DNA Part A* 27**,** 1001-1002.

Sissini, M.N., Navarrete-Fernández, T.M., Murray, E.M.C., Freese, J.M., Gentilhomme, A.S., Huber, S.R., Mumford, T.F., and Hughey, J.R. (2016). Mitochondrial and plastid genome analysis of the heteromorphic red alga *Mastocarpus papillatus* (C. Agardh) Kützing (Phyllophoraceae, Rhodophyta) reveals two characteristic florideophyte organellar genomes. *Mitochondrial DNA Part B* 1, 676-677.

Song, S.L., Yong, H.S., Lim, P.E., and Phang, S.M. (2017). Complete mitochondrial genome of *Gracilaria changii* (Rhodophyta: Gracilariaceae). *J. Appl. Phycol.* 29**,** 2129-2134.

Tablizo, F.A., and Lluisma, A.O. (2014). The mitochondrial genome of the red alga *Kappaphycus striatus* ("Green Sacol" variety): Complete nucleotide sequence, genome structure and organization, and comparative analysis. *Mar. Genomics* 18**,** 155-161.

Tamayo, D.A., and Hughey, J.R. (2016). Organellar genome analysis of the marine red alga *Dasya binghamiae* (Dasyaceae, Rhodophyta) reveals an uncharacteristic florideophyte mitogenome structure. *Mitochondrial DNA Part B* 1, 510-511.

Tang, X., Jia, X., Zhang, J., and Liu, T. (2019). The complete mitogenome of *Gracilaria chouae* and its phylogenetic analysis. *Mitochondrial DNA Part B* 4**,** 2786-2787.

Tang, X., Li, Y., Chen, W.Z., Liu, T., and Zhang, J. (2018). Complete sequence of mitochondrial DNA of *Gracilaria tenuistipitata* var. *liui* (Rhodophyta). *Mitochondrial DNA Part B* 3, 1203-1204.

Wang, L., Mao, Y., Kong, F., Li, G., Ma, F., Zhang, B., Sun, P., Bi, G., Zhang, F., Xue, H., and Cao, M. (2013). Complete sequence and analysis of plastid genomes of two economically important red algae: *Pyropia haitanensis* and *Pyropia yezoensis*. *PLoS One* 8, e65902.

Wang, S., Zhang, J., Qian, H., Chen, W., Liu, T., and Tang, X. (2019). Complete mitochondrial genome and phylogenetic analysis of *Gracilaria spinulosa*. *Mitochondrial DNA Part B* 4, 2046-2047.

Watanabe, K., Kishimoto, T., Kumagai, Y., Shimizu, T., Uji, T., Yasui, H., and Kishimura, H. (2019). Complete sequence of mitochondrial DNA of *Gloiopeltis furcata* (Postels and Ruprecht) J. Agardh. *Mitochondrial DNA Part B* 4, 2543-2544.

Williamson, C., Yesson, C., Briscoe, A.G., and Brodie, J. (2016). Complete mitochondrial genome of the geniculate calcified red alga, *Corallina officinalis* (Corallinales, Rhodophyta). *Mitochondrial DNA Part B* 1, 326-327.

Wolf, D.I., Evans, J.R., and Vis, M.L. (2017). Complete mitochondrial genome of the freshwater red alga *Lympha mucosa* (Rhodophyta). *Mitochondrial DNA Part B* 2, 707-708.

Xu, K., Tang, X., Bi, G., Cao, M., Wang, L., and Mao, Y. (2018). The first complete organellar genomes of an Antarctic red alga, *Pyropia endiviifolia*: insights into its genome architecture and phylogenetic position within genus *Pyropia* (Bangiales, Rhodophyta). *J. Oceanol. Limnol.* 36, 1315-1328.

Xu, K., Yu, X., Tang, X., Kong, F., and Mao, Y. (2019). Organellar genome variation and genetic diversity of Chinese *Pyropia yezoensis*. *Front. Mar. Sci.* 6, 75.

Yang, E.C., Kim, K.M., Boo, G.H., Lee, J.H., Boo, S.M., and Yoon, H.S. (2014a). Complete mitochondrial genome of the agarophyte red alga *Gelidium vagum* (Gelidiales). *Mitochondrial DNA* 25, 267-268.

Yang, E.C., Kim, K.M., Kim, S.Y., Lee, J., Boo, G.H., Lee, J.H., Nelson, W.A., Yi, G., Schmidt, W.E., Fredericq, S., Boo, S.M., Bhattacharya, D., and Yoon, H.S. (2015). Highly conserved mitochondrial genomes among multicellular red algae of the Florideophyceae. *Genome Biol. Evol.* 7, 2394-2406.

Yang, E.C., Kim, K.M., Kim, S.Y., and Yoon, H.S. (2014b). Complete mitochondrial genome of agar-producing red alga *Gracilariopsis chorda* (Gracilariales). *Mitochondrial DNA* 25, 339-341.

Yesson, C., Bian, X., Williamson, C., Briscoe, A.G., and Brodie, J. (2020). Mitochondrial and plastid genome variability of *Corallina officinalis* (Corallinales, Rhodophyta). *Applied Phycology* 1, 73-79.

Yuan, X. (2018). The complete mitochondrial genome of *Gracilaria textorii* (Gracilariales, Florideophyceae). *Mitochondrial DNA Part B* 3, 438-439.

Zhang, J., Tang, X., Zhou Chen, W., Liu, T., and Li, Y. (2018). The complete plastid genome of *Grateloupia filicina* (Rhodophyta) and phylogenetic analysis. *Mitochondrial DNA Part B* 3, 1172-1173.

Zhang, Y., Guo, Y.-M., Li, T.J., Chen, C.H., Shen, K.N., and Hsiao, C.D. (2016). The complete chloroplast genome of *Gracilariopsis lemaneiformis*, an important economic red alga of the family Gracilariaceae. *Mitochondrial DNA Part B* 1, 2-3.
